# Supplementary material for: Selective PROTAC-mediated degradation of SMARCA2 is efficacious in SMARCA4 mutant cancers
Source: Nat Commun. 2022 Nov 10;13:6814. doi: 10.1038/s41467-022-34562-5 (PMC9649729; doi:10.1038/s41467-022-34562-5)
Supplement: Supplementary file 1 — Supplementary Information [file 41467_2022_34562_MOESM1_ESM.pdf]

Supplementary Information

Supplementary Table 1. UniProtKB annotation of SMARCA2 and SMARCA4 isoforms

Gene	UniProt	UniProt isoform	NCBI Isoform definition	Difference from canonical sequence	AA length	NCBI mRNA Reference	NCBI reference sequence
SMARCA2	P51531-1	isoform 1	isoform a		1590	NM_003070	NP_003061.3
SMARCA2	P51531-2	isoform 2	isoform b	1400-1417: Missing	1572	NM_139045.3	NP_620614.2
SMARCA4	P51532-1	isoform 1	isoform B		1647	NM_003072.3	NP_003063.2
SMARCA4	P51532-2	isoform 2	isoform E	1259-1291: Missing.	1614	NM_001128847.1	NP_001122319.1
SMARCA4	P51532-3	isoform 3	isoform D	1259-1291: Missing; 1388-1388: W → WLKT; 1475-1475: Missing.	1616	NM_001128846.1	NP_001122318.1
SMARCA4	P51532-4	isoform 4	isoform C	1259-1291: Missing; 1388-1388: W → WLKT	1617	NM_001128845.1	NP_001122317.1
SMARCA4	P51532-5	isoform 5	isoform F	1259-1291: Missing; 1475-1475: Missing.	1613	NM_001128848.1	NP_001122320.1

Supplementary Figure 1.

a

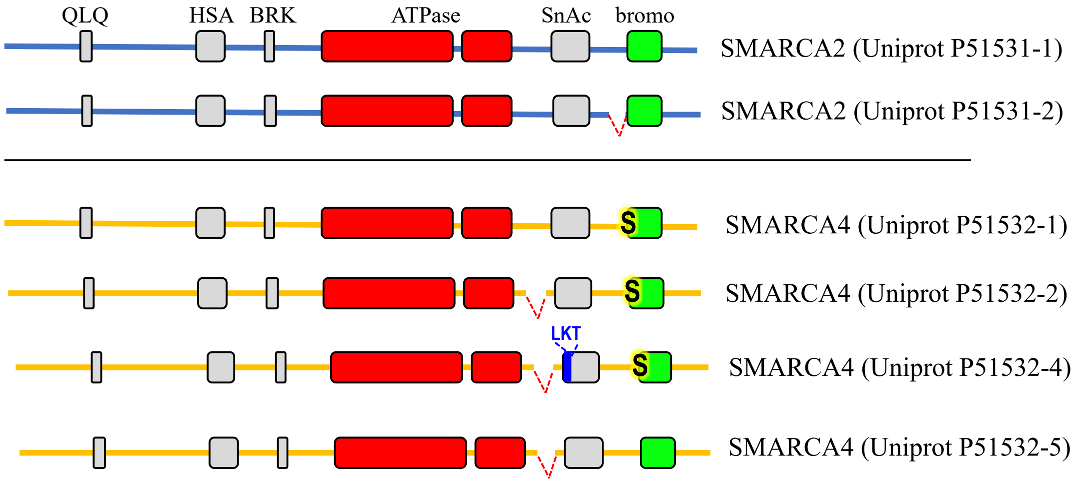

b

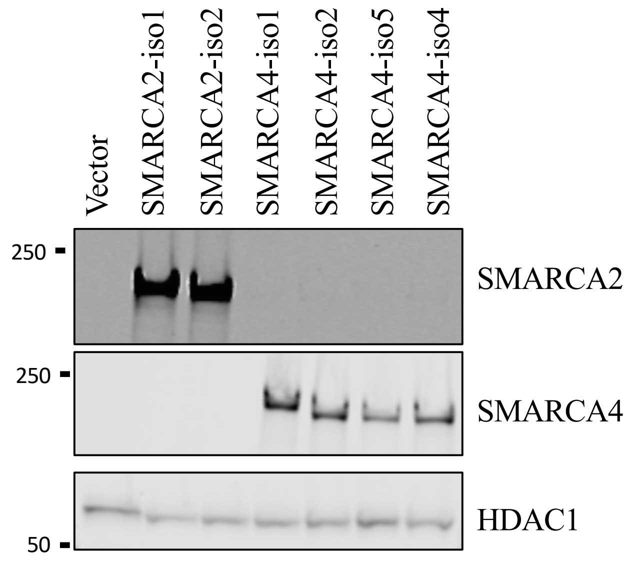

Supplementary Figure 1. a, Schematic of SMARCA2 and SMARCA4 isoforms generated for ectopic expression studies. SMARCA4 isoform 3 (P51532-3) was not generated due to the fact that the amino acid differences annotated for this isoform were captured in the other SMARCA4 isoforms. **b,** Immunblot analysis of SMARCA2 and SMARCA4 isoform expression in human TOV112D cells that are deficient in endogenous SMARCA4. HDAC1 serves as a loading control. Results were confirmed in 3 similar experiments.

Supplementary Figure 2.

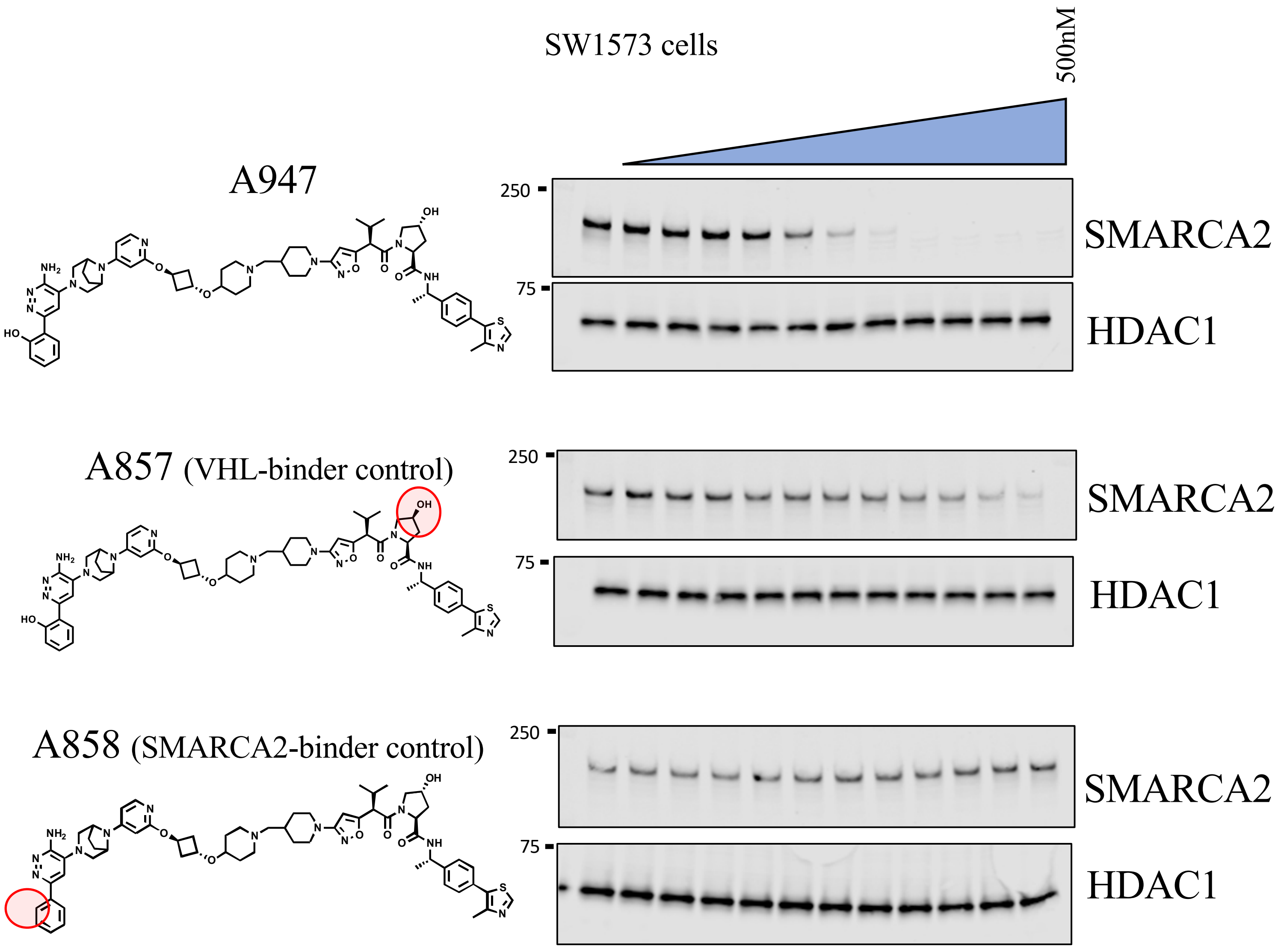

Supplementary Figure 2. Immunblot analysis of SMARCA2 following 18h treatment of SW1573 cells with A947 and control molecules defective in binding to SMARCA2/A4 (A858) and VHL (A857). HDAC1 serves as a loading control. The chemical structures of the respective molecules are shown, with modification sites highlighted in red. Data are representative of 2 similar experiments.

Supplementary Figure 3

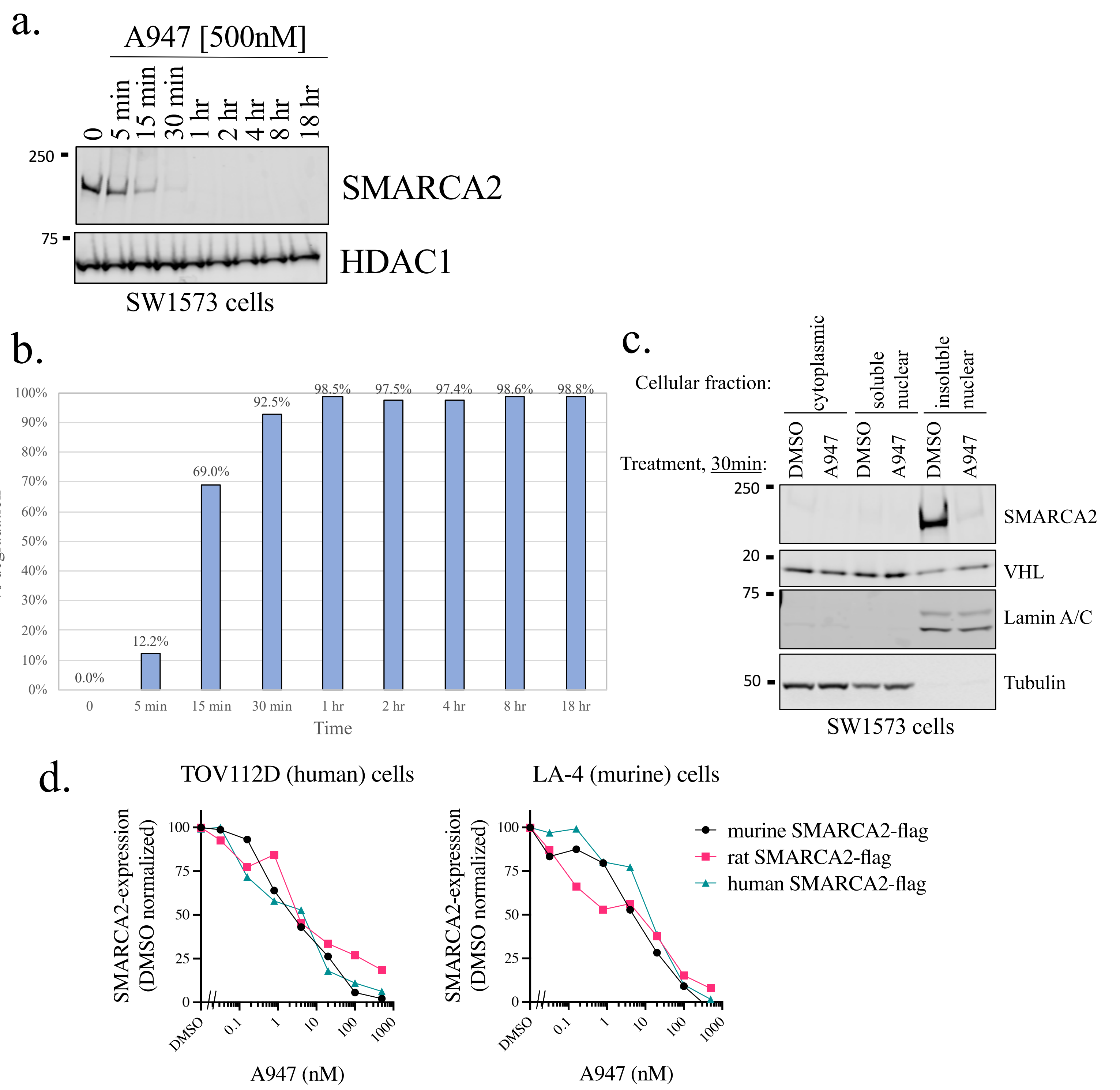

Supplementary Figure 3. a, Timecourse of A947 treatment on SMARCA2 protein levels in SW1573 cells. Data is representative of one experiment. **b,** SMARCA2 immublots were quantified by Licor and presented as a percent degradation relative to the untreated control lysates. **c**, Immunoblot analysis of cytoplasmic, nuclear soluble and nuclear insoluble fractions of SW1573 evaluating SMARCA2 and VHL protein levels following 30min treatment with A947 (100nM). Lamin served as a control for the nuclear insoluble fraction and tubulin for the cytoplasmic fraction. Cellular fractionation was carried out as described in the Methods. Data is representative of two independent experiments. **d,** Licor-based quantification of SMARCA2 levels in human (TOV112D) and murine (LA4) cell lines expressed SMARCA2 orthologs upon 24h treatment with a dose-response of A947. Data is normalized to levels of the respective SMARCA2 ortholog in control (DMSO) lysates. Data in a, c are representive of 2 independent experiments.

Supplementary Figure 4.

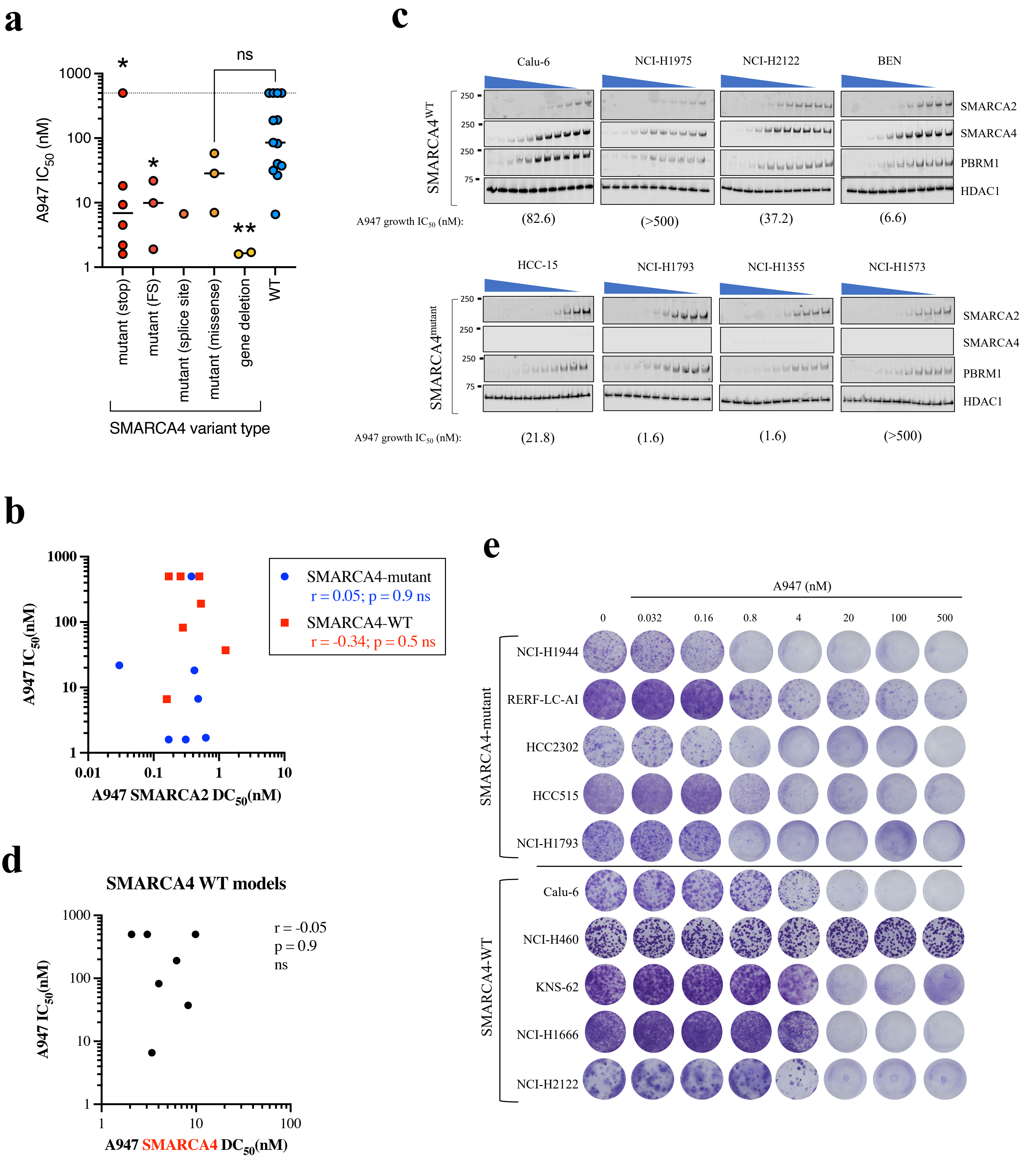

Supplementary Figure 4. a, Effect of A947 treatment on the growth of 28 lung cancer cell lines classified by *SMARCA4* gene mutation variant types and data is represented as the concentration of A947 required to inhibit growth by 50% (IC_{50}) following 7 days of treatment. Individual cell line IC_{50}'s were determined from n=3 biologic replicates. Median IC_{50}'s across models defined by variant types are indicated by the black line. FS, frameshift. Significance was assessed by a two-tailed, Mann Whitney test. *p<0.05 (stop mutation p=0.021, frameshift p=0.014), **p=0095, ns, not significant. **b,** Scatterplots demonstrating lack of an association between growth inhibition (y-axis, IC_{50}) with degradation of SMARCA2 (x-axis, DC_{50}), as determined by immunofluorescence, in $SMARCA4^{mut}$ (n=7) and $SMARCA4^{WT}$ (n=7) models. Statistical significance was assessed by a two-sided, unpaired Student t-test; p=0.52, non-significant. In addition, lack of correlation within $SMARCA4^{mut}$ and $SMARCA4^{WT}$ cell lines was calculated by the Pearson coefficient. **c,** Immunblot analysis of SMARCA2, SMARCA4 and PBRM1 levels following 18h treatment of cells with A947. Cell lines were chosen due to differential growth inhibition, as indicated by the IC_{50} measurements below the immunoblots. HDAC1 serves as a loading control. Data were confirmed in a similar experiment. **d,** Scatterplots demonstrating lack of an association between growth inhibition (y-axis, IC_{50}) with degradation of SMARCA4 (x-axis, DC_{50}), as determined by immunofluorescence, in $SMARCA4^{WT}$ models (n=7). Lack of correlation was calculated by the Pearson coefficient. **e,** Effect of a dose-response of A947 on clonogenic growth of a panel of 10 lung cancer cell lines defined by SMARCA4 mutation status.

Supplementary Figure 5.

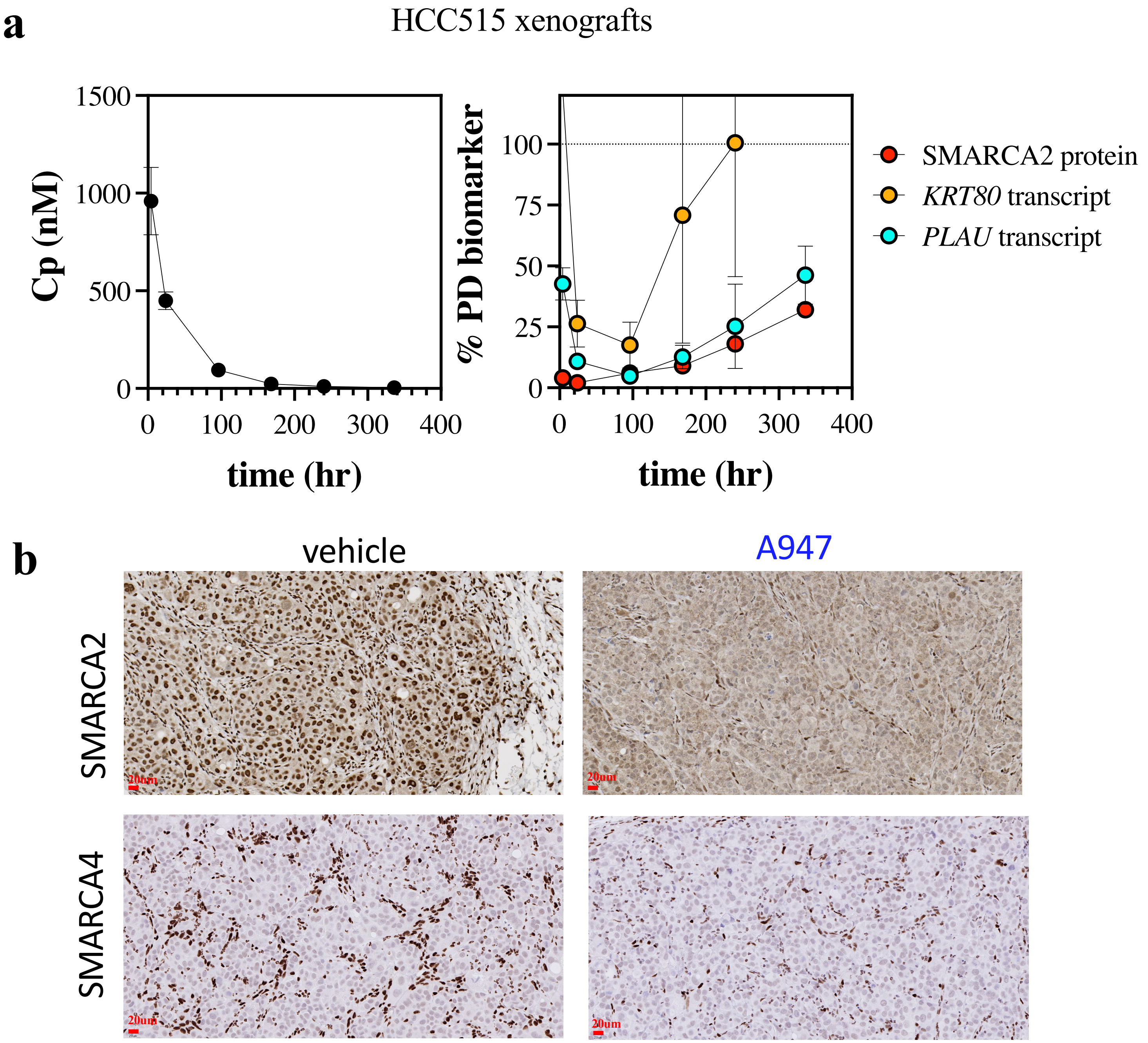

Supplementary Figure 5. a, Plasma concentration (left graphic) and pharmacodynamic biomarker responses (right graphic) monitored over a two week period in HCC515 xenografts following a single-dose, intravenous (i.v.) administration of A947 (40mg/kg). Tumor levels of SMARCA2 protein were quantified by ImageLab from Western blots and normalized to a loading control protein (β-actin). Tumor levels of the mRNA transcripts, *KRT80* and *PLAU*, were quantified by Taqman. Data is presented relative to levels in untreated tumors and represented as mean ± s.d from n=5 animals per timepoint. **b,** Immunhistologic assessment of SMARCA2 and SMARCA4 levels in SMARCA4^{mut} HCC515 xenografts following administration (40mg/kg, i.v.) of A947 or vehicle control for 24h. Images are representative of n=5 tumors/group. Scale bar = 20um. Note the SMARCA4 signal localized to the stromal infiltrate in this SMARCA4 mutant model.

Supplementary Figure 6.

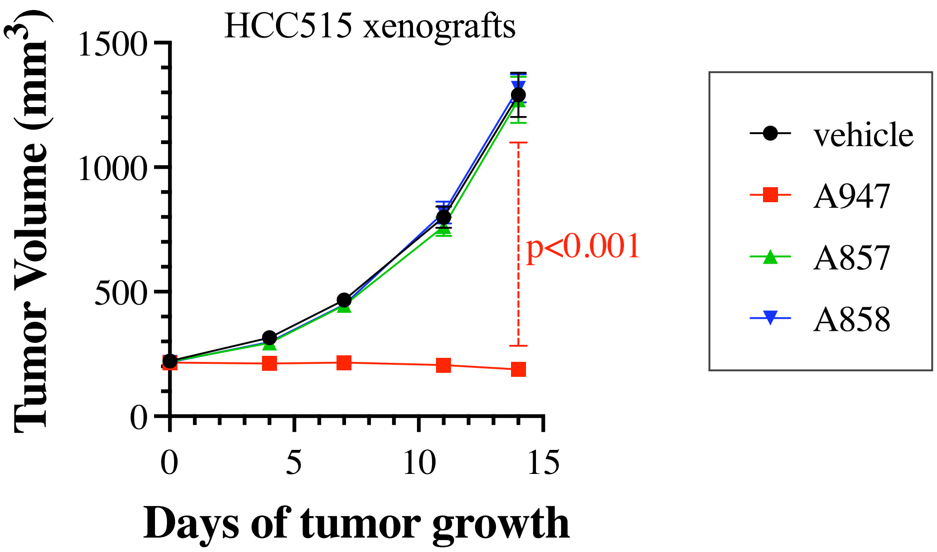

Supplemental Figure 6. Tumor volume in mice harboring either SMARCA4-mutant HCC515 xenografts following administration of A947 or control analogs defective in VHL (A857) and SMARCA2 (A858) binding (40mg/kg, i.v., all groups). Data is presented as mean ± s.e.m. (n=8 mice / group). Statistical significance was assessed by a two-sided, unpaired Student t-test, p=3.8e-8.

Supplementary Figure 7.

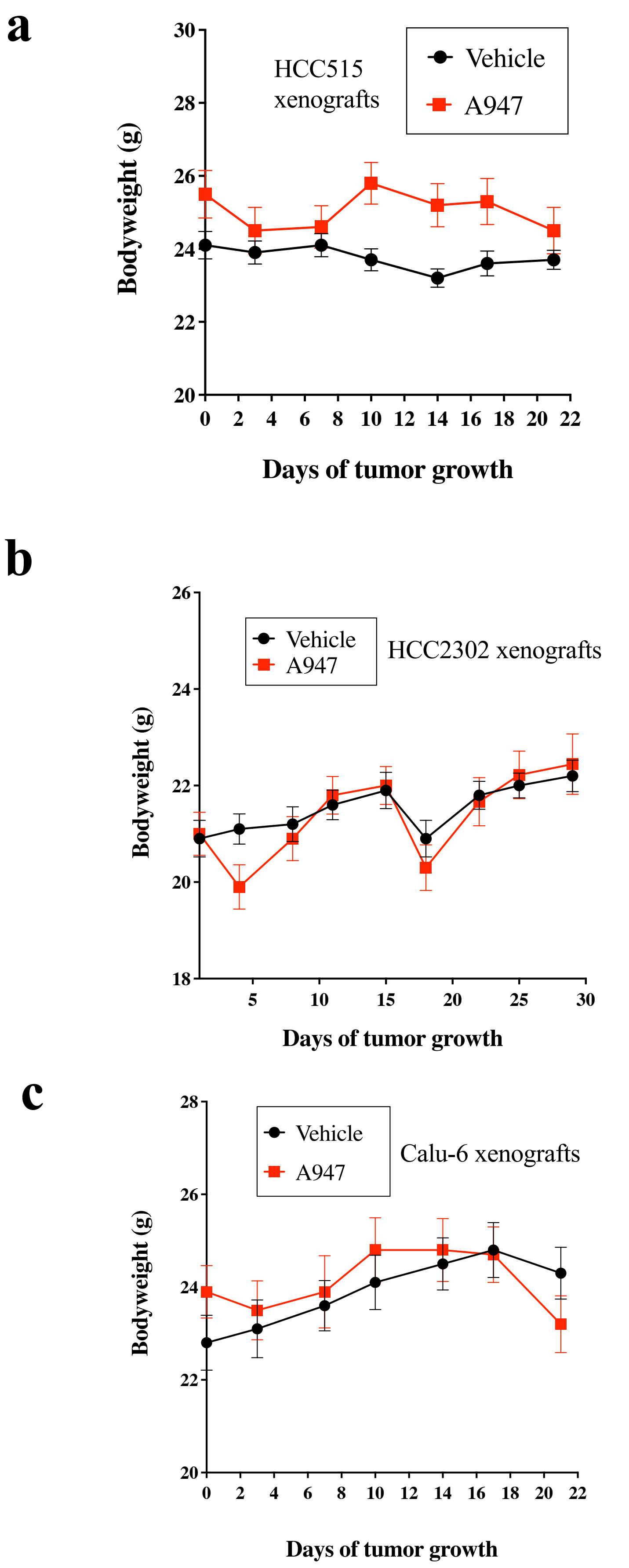

Supplemental Figure 7. Body weight of mice harboring either SMARCA4-mutant HCC515 **(a),** HCC2302 **(b)** or Calu-6 **(c)** xenografts following administration (40mg/kg, i.v.) of A947 or vehicle control. Data is presented as mean ± s.e.m. (n=10 mice / group). Statistical significance of the A947 treated group was assessed by a two-sided, unpaired Student t-test.

Supplementary Figure 8.

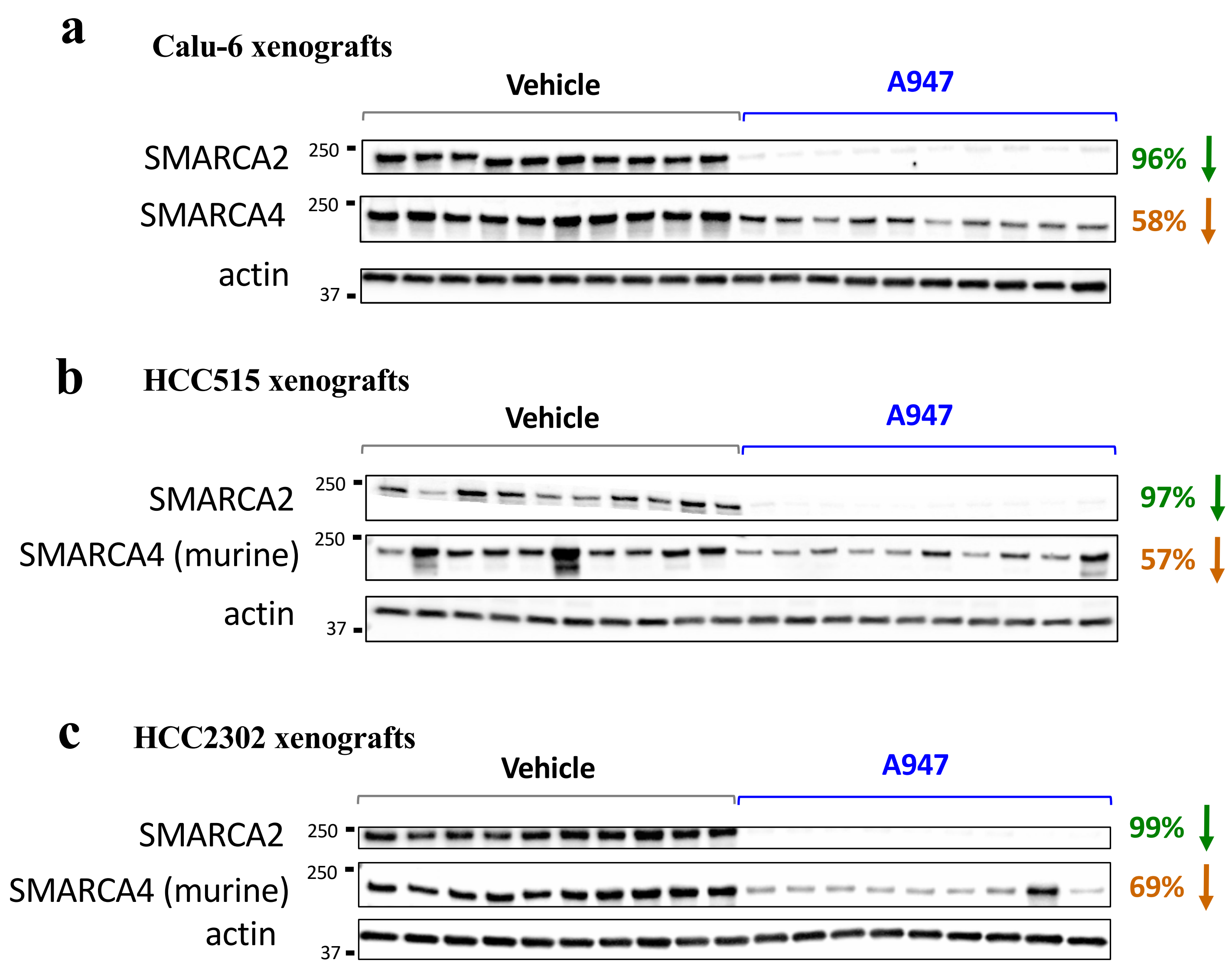

Supplemental Figure 8. Immunoblot analysis of Calu-6 **(a)**, HCC515 **(b)** and HCC2302 **(c)** tumors collected at end of study from animals treated in Fig. 4f, Fig. 4b, & Fig. 4c, respectively. Mice received a final dose of A947 24 hours prior to tumor collection. In SMARCA4^{mut} xenografts, HCC515 and HCC2302, the SMARCA4 signal represents the murine stromal infiltrate within these SMARCA4-mutant human xenografts. β-actin served as a loading control. The A947-mediated decrease in the protein signal relative to the vehicle control was determined through ImageLab-quantification of b-actin-normalized protein signal. N=10 tumors/group with the exception of HCC2302, A947-treated xenografts (n=9). Data is representative of a minimum of two independent experiments for each model.

Supplementary Figure 9.

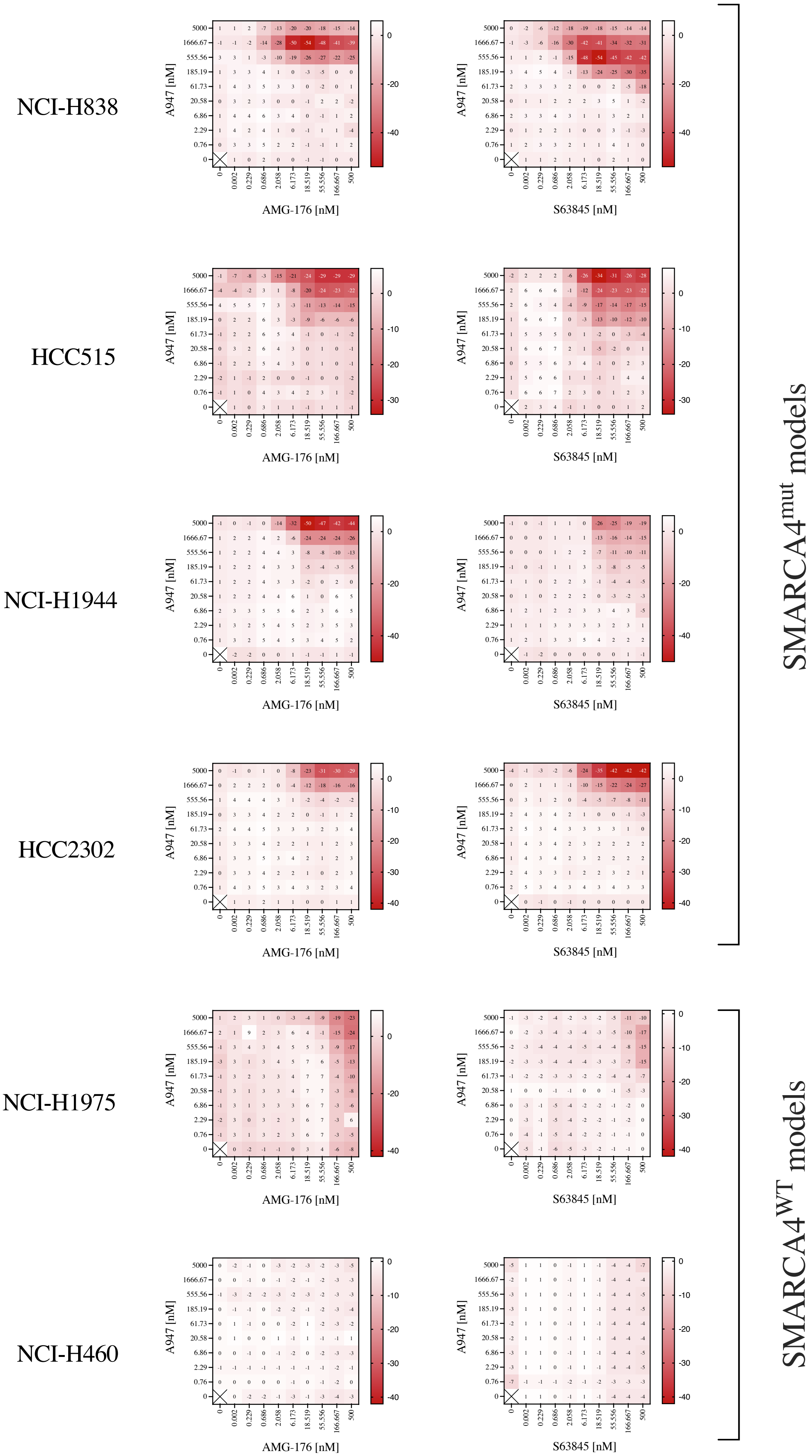

Supplemental Figure 9. Treatment of 4 additional SMARCA4-mutant cell lines and 2 SMARCA4-WT cell lines with a 9x9 matrix titration of A947 with the MCL1 inhibitors, AMG-176 (left plots) and S63845 (right plots). Heatmaps depict activity in excess of the Bliss independence model to describe synergistic drug interactions.

Supplementary Note: Chemical Synthesis

All chemicals, reagents and solvents were at least reagent grade and obtained from various commercial sources. General removal of solvents was conducted by using a rotary evaporator. Residual solvents were then removed from the compounds using a vacuum manifold with < 10 mm Hg pressure and/or via compound lyophilization. NMR spectra were recorded on a Bruker 400 MHz spectrometer and are reported in parts per million (ppm) using residual nondeuterated solvents. LCMS spectral data was obtained using standard electrospray ionization mass spectrometry. Normal phase silica gel chromatography, flash column and preparatory thin-layer, was conducted on Merck silica gel 60, or as specifically indicated. Reverse phase and chiral chromatographic separations were conducted using columns and conditions specifically indicated in the experimental procedures below.

4-((1r,3r)-3-(Benzyloxy)cyclobutoxy)pyridine (**1**)
To a solution of pyridin-4-ol (3.20 g, 33.7 mmol, 1.5 *eq*) and (1s,3s)-3-(benzyloxy)cyclobutan-1-ol (4 g, 22.4 mmol, 1 *eq*) in tetrahydrofuran (200 mL) was added triphenylphosphine (7.06 g, 26.9 mmol, 1.2 *eq*) and diisopropyl azodicarboxylate (5.45 g, 26.9 mmol, 1.2 *eq*) in one portion at 10 °C under nitrogen. The mixture was stirred at 50 °C for 12 hours. The reaction mixture was concentrated under reduced pressure to remove tetrahydrofuran. Water (50 mL) was added, and the mixture was stirred for 1 minute. The aqueous phase was extracted with dichloromethane (50 mL x 3). The combined organic phase was washed with brine (50 mL x 2), dried with anhydrous sodium sulfate, filtered, and concentrated in vacuum. The residue was purified by silica gel column chromatography (petroleum ether: tetrahydrofuran from 20:1 to 5:1) and, additionally, by reverse phase C18 column chromatography [5–50% acetonitrile: water (0.5% ammonium hydroxide)]. 4-((1r,3r)-3-(Benzyloxy)cyclobutoxy)pyridine (3.2 g, 12.5 mmol, 55% yield) was obtained as a white solid.

1-Benzyl-4-((1r,3r)-3-(benzyloxy)cyclobutoxy)pyridin-1-ium bromide (**2**)
To a solution of 4-((1r,3r)-3-(Benzyloxy)cyclobutoxy)pyridine (4.2 g, 16.5 mmol, 1 *eq*) in toluene (65 mL) was added benzyl bromide (2.81 g, 16.5 mmol, 1 *eq*). The mixture was stirred at 80 °C for 12 hours. The reaction mixture was concentrated under reduced pressure to remove toluene. The crude product was triturated with petroleum ether (80 mL). 1-Benzyl-4-((1r,3r)-3-(benzyloxy)cyclobutoxy)pyridin-1-ium bromide (6.5 g, 15.3 mmol, 92% yield) was obtained as a white solid.

1-Benzyl-4-((1r,3r)-3-(benzyloxy)cyclobutoxy)-1,2,3,6-tetrahydropyridine (**3**)
To a solution of 1-benzyl-4-((1r,3r)-3-(benzyloxy)cyclobutoxy)pyridin-1-ium bromide (6.5 g, 15.3 mmol, 1 *eq*) in ethanol (120 mL) was added sodium borohydride (3.46 g, 91.5 mmol, 6 *eq*) at 0 °C. The mixture was stirred at 15 °C for 4 hours. The reaction mixture was concentrated under reduced pressure to remove ethanol. The residue was diluted with water (25 mL) and extracted with ethyl acetate (50 mL x 2). The combined organic phase was washed with saturated brine (40 mL x 3), dried with anhydrous sodium sulfate, filtered, and concentrated in vacuum. 1-Benzyl-4-((1r,3r)-3-(benzyloxy)cyclobutoxy)-1,2,3,6-tetrahydropyridine (4.5 g, 12.9 mmol, 84% yield) was obtained as a colorless oil.

(1r,3r)-3-((1-Benzylpiperidin-4-yl)oxy)cyclobutan-1-ol (**4**)
To a solution of 1-benzyl-4-((1r,3r)-3-(benzyloxy)cyclobutoxy)-1,2,3,6-tetrahydropyridine (4.5 g, 12.9 mmol, 1 *eq*) in tetrahydrofuran (95 mL) and ethanol (70 mL) was added 10% palladium on activated carbon catalyst (0.5 g) under nitrogen atmosphere. The suspension was degassed and purged with hydrogen three times. The mixture was stirred under hydrogen pressure (50 psi) at 35 °C for 12 hours. The reaction mixture was filtered, and the filtrate was concentrated. The residue was purified by silica gel column chromatography (dichloromethane: methanol: ammonium hydroxide from 20:1:0 to 10:1:0.1). (1r,3r)-3-((1-Benzylpiperidin-4-yl)oxy)cyclobutan-1-ol (2.8 g, 10.71 mmol, 83% yield) was obtained as a colorless oil.

tert-Butyl 4-((1r,3r)-3-hydroxycyclobutoxy)piperidine-1-carboxylate (**5**)
To a solution of (1r,3r)-3-((1-benzylpiperidin-4-yl)oxy)cyclobutan-1-ol (1.1 g, 4.21 mmol, 1 *eq*) in methanol (10 mL) was added 10% palladium hydroxide on activated carbon catalyst (591 mg) and di-*tert*-butyl dicarbonate (1.84 g, 8.42 mmol, 2 *eq*) under nitrogen atmosphere. The suspension was degassed and purged with hydrogen three times. The mixture was stirred under hydrogen pressure (50 psi) at 25 °C for 12 hours. The reaction mixture was filtered, and the filtrate was concentrated. The residue was purified by silica gel chromatography (petroleum ether:

ethyl acetate from 20:1 to 2:1). *tert*-Butyl 4-((1r,3r)-3-hydroxycyclobutoxy)piperidine-1-carboxylate (820 mg, 3.02 mmol, 71% yield) was obtained as a colorless oil.

tert-Butyl 4-((1r,3r)-3-((4-bromopyridin-2-yl)oxy)cyclobutoxy)piperidine-1-carboxylate (**6**)
A mixture of *tert*-butyl 4-((1r,3r)-3-hydroxycyclobutoxy)piperidine-1-carboxylate (1 g, 3.69 mmol, 1 *eq*), 4-bromo-2-fluoro-pyridine (778 mg, 4.42 mmol, 1.2 *eq*), and cesium carbonate (2.40 g, 7.37 mmol, 2 *eq*) in acetonitrile (10 mL) was degassed and purged with nitrogen three times. The mixture was then stirred at 90 °C for 12 hours under the nitrogen atmosphere. The reaction mixture was filtered and concentrated. The crude product was purified by silica gel chromatography (petroleum ether: ethyl acetate from 1:0 to 10:1). *tert*-Butyl 4-((1r,3r)-3-((4-bromopyridin-2-yl)oxy)cyclobutoxy)piperidine-1-carboxylate (1.36 g, 3.18 mmol, 86% yield) was obtained as colorless oil.

Benzyl 8-(2-((1r,3r)-3-((1-(*tert*-butoxycarbonyl)piperidin-4-yl)oxy)cyclobutoxy)pyridin-4-yl)-3,8-diazabicyclo[3.2.1]octane-3-carboxylate (**7**)
A mixture of benzyl 3,8-diazabicyclo[3.2.1]octane-3-carboxylate (784 mg, 3.18 mmol, 1 *eq*), *tert*-butyl 4-((1r,3r)-3-((4-bromopyridin-2-yl)oxy)cyclobutoxy)piperidine-1-carboxylate (1.36 g, 3.18 mmol, 1 *eq*), chloro(2-dicyclohexylphosphino-2',6'-diisopropoxy-1,1'-biphenyl)[2-(2'-amino-1,1'-biphenyl)]palladium(II) [RuPhos-Pd-G2] (148 mg, 0.19 mmol, 0.06 *eq*), and cesium carbonate (2.07 g, 6.37 mmol, 2 *eq*) in toluene (27 mL) was degassed and purged with nitrogen three times. The mixture was then stirred at 110 °C for 12 h under the nitrogen atmosphere. Water (30 mL) was added, and the mixture was extracted with ethyl acetate (40 mL x 2). The combined organic layers were washed with saturated aqueous sodium chloride (30 mL x 2), dried over anhydrous sodium sulfate, filtered and concentrated under reduced pressure. The crude product was purified by silica gel chromatography (petroleum ether: ethyl acetate from 1:0 to 1:1). Benzyl 8-(2-((1r,3r)-3-((1-(*tert*-butoxycarbonyl)piperidin-4-yl)oxy)cyclobutoxy)pyridin-4-yl)-3,8-diazabicyclo[3.2.1]octane-3-carboxylate (1.35 g, 2.28 mmol, 71% yield) was obtained as a colorless oil.

tert-Butyl 4-((1r,3r)-3-((4-(3,8-diazabicyclo[3.2.1]octan-8-yl)pyridin-2-yl)oxy)cyclobutoxy)piperidine-1-carboxylate (**8**)
To a solution of benzyl 8-(2-((1r,3r)-3-((1-(*tert*-butoxycarbonyl)piperidin-4-yl)oxy)cyclobutoxy)pyridin-4-yl)-3,8-diazabicyclo[3.2.1]octane-3-carboxylate (1.35 g, 2.28 mmol, 1 *eq*) in a mixture of tetrahydrofuran (27 mL) and ethanol (27 mL) was added 10% palladium hydroxide on activated carbon catalyst (320 mg) under nitrogen. The suspension was degassed under vacuum and purged with hydrogen several times. The mixture was stirred under hydrogen pressure (50 psi) at 60 °C for 12 hours. The reaction mixture was filtered, and the filtrate was concentrated. *tert*-Butyl 4-((1r,3r)-3-((4-(3,8-diazabicyclo[3.2.1]octan-8-yl)pyridin-2-yl)oxy)cyclobutoxy)piperidine-1-carboxylate (932 mg, 2.03 mmol, 89% yield) was obtained as a brown solid.

tert-Butyl 4-((1r,3r)-3-((4-(3-(3-amino-6-chloropyridazin-4-yl)-3,8-diazabicyclo[3.2.1]octan-8-yl)pyridin-2-yl)oxy)cyclobutoxy)piperidine-1-carboxylate (**9**)
A mixture of *tert*-butyl 4-((1r,3r)-3-((4-(3,8-diazabicyclo[3.2.1]octan-8-yl)pyridin-2-yl)oxy)cyclobutoxy)piperidine-1-carboxylate (932 mg, 2.03 mmol, 1 *eq*), 4-bromo-6-chloro-pyridazin-3-amine (508 mg, 2.44 mmol, 1.2 *eq*), and *N*,*N*-diisopropylethylamine (2.63 g, 20.3 mmol, 3.54 mL, 10 *eq*) in dimethylsulfoxide (30 mL) was degassed and purged with nitrogen three times. The mixture was then stirred at 130 °C for 3 h under the nitrogen atmosphere. Water (30 mL) was added, and the mixture was extracted with ethyl acetate (30 mL x 2). The combined organic layers were washed saturated aqueous sodium chloride (30 mL x 2), dried over anhydrous sodium sulfate and concentrated. The residue was purified by semi-preparative reverse phase HPLC (column: Phenomenex Gemini C18 250*50 10 um; mobile phase: [water (0.05% ammonia hydroxide v/v)-ACN]; B%: 45%-70%). *tert*-Butyl 4-((1r,3r)-3-((4-(3-(3-amino-6-chloropyridazin-4-yl)-3,8-diazabicyclo[3.2.1]octan-8-yl)pyridin-2-yl)oxy)cyclobutoxy)piperidine-1-carboxylate (772 mg, 1.32 mmol, 64% yield) was obtained as a yellow oil.

tert-Butyl 4-((1r,3r)-3-((4-(3-(3-amino-6-(2-hydroxyphenyl)pyridazin-4-yl)-3,8-diazabicyclo[3.2.1]octan-8-yl)pyridin-2-yl)oxy)cyclobutoxy)piperidine-1-carboxylate (10)
A mixture of *tert*-butyl 4-((1r,3r)-3-((4-(3-(3-amino-6-chloropyridazin-4-yl)-3,8-diazabicyclo[3.2.1]octan-8-yl)pyridin-2-yl)oxy)cyclobutoxy)piperidine-1-carboxylate (772 mg, 1.32 mmol, 1 *eq*), (2-hydroxyphenyl)boronic acid (218 mg, 1.58 mmol, 1.2 *eq*), tetrakis[triphenylphosphine]palladium(0) (152 mg, 0.13 mmol, 0.1 *eq*), and potassium carbonate (364 mg, 2.63 mmol, 2 *eq*) in a mixture of dioxane (12 mL) and water (2 mL) was degassed and purged with nitrogen three times. The mixture was then stirred at 90 °C for 10 h under the nitrogen atmosphere.

The reaction mixture was concentrated. The residue was purified by semi-preparative reverse phase HPLC (column: Kromasil 250*50 mm*10 um; mobile phase: [water (0.1%trifluoroacetic acid)-ACN]; B%: 20%-50%). *tert*-Butyl 4-((1r,3r)-3-((4-(3-(3-amino-6-(2-hydroxyphenyl)pyridazin-4-yl)-3,8-diazabicyclo[3.2.1]octan-8-yl)pyridin-2-yl)oxy)cyclobutoxy)piperidine-1-carboxylate (500 mg, 0.71 mmol, 54% yield) was obtained as a yellow solid.

2-(6-amino-5-(8-(2-((1r,3r)-3-(piperidin-4-yloxy)cyclobutoxy)pyridin-4-yl)-3,8-diazabicyclo[3.2.1]octan-3-yl)pyridazin-3-yl)phenol (**11**)

To a solution of *tert*-butyl 4-((1r,3r)-3-((4-(3-(3-amino-6-(2-hydroxyphenyl)pyridazin-4-yl)-3,8-diazabicyclo[3.2.1]octan-8-yl)pyridin-2-yl)oxy)cyclobutoxy)piperidine-1-carboxylate (500 mg, 0.78 mmol, 1 *eq*) in dichloromethane (5 mL) was added hydrochloric acid/dioxane (4 M, 5 mL, 25.8 *eq*). The mixture was stirred at 20 °C for 2 hours. The mixture was concentrated, and crude 2-(6-amino-5-(8-(2-((1r,3r)-3-(piperidin-4-yloxy)cyclobutoxy)pyridin-4-yl)-3,8-diazabicyclo[3.2.1]octan-3-yl)pyridazin-3-yl)phenol trihydrochloride (500 mg) was obtained as a yellow solid.

Methyl 2-(3-hydroxyisoxazol-5-yl)-3-methyl-butanoate (**12**)

To a solution of 2-(3-hydroxyisoxazol-5-yl)-3-methyl-butanoic acid[1] (1 g, 5.40 mmol, 1 *eq*) in methanol (10 mL) was added thionyl chloride (2.57 g, 21 mmol, 1.57 mL, 4 *eq*) at 0°C. The reaction mixture was stirred at 70 °C for 3 hours. The reaction mixture was concentrated under reduced pressure. The residue was diluted with water (50 ml) and extracted with ethyl acetate (30 mL x 3). The combined organic layers were washed with brine (80 mL x 2), dried over anhydrous sodium sulfate, filtered, and concentrated under reduced pressure. Methyl 2-(3-hydroxyisoxazol-5-yl)-3-methyl-butanoate (1 g, 5.02 mmol, 92% yield) was obtained as a yellow oil.

Methyl 3-methyl-2-[3-(1,1,2,2,3,3,4,4,4-nonafluorobutylsulfonyloxy)isoxazol-5-yl]butanoate (**13**)

To a solution of methyl 2-(3-hydroxyisoxazol-5-yl)-3-methyl-butanoate (800 mg, 4.02 mmol, 1 *eq*) in acetonitrile (5 mL) was added potassium carbonate (1.11 g, 8.03 mmol, 2 *eq*) and perfluorobutyl sulfonyl fluoride (1.46 g, 4.82 mmol, 1.2 *eq*). The reaction mixture was stirred at 25°C for 12 hours. The reaction mixture was diluted with water (50 mL) and extracted with ethyl acetate (30 mL x 3). The combined organic layers were washed with brine (80 mL x 3), dried over anhydrous sodium sulfate, filtered, and concentrated under reduced pressure. The residue was purified by silica gel column chromatography (petroleum ether/ethyl acetate from 100:1 to 20:1). Methyl 3-methyl-2-[3-(1,1,2,2,3,3,4,4,4-nonafluorobutylsulfonyloxy)isoxazol-5-yl]butanoate (530 mg, 1.10 mmol, 27 % yield) was obtained as a colorless oil.

Methyl 2-[3-[4-(dimethoxymethyl)-1-piperidyl] isoxazol-5-yl]-3-methyl-butanoate (**14**)

To a mixture of methyl 3-methyl-2-[3-(1,1,2,2,3,3,4,4,4-nonafluorobutylsulfonyloxy)isoxazol -5-yl]butanoate (5 g, 10.39 mmol, 1 *eq*) and 4-(dimethoxymethyl)piperidine (4.14 g, 26.0 mmol, 2.5 *eq*) in *N,N*-dimethylformamide (50 mL) was added *N,N*-diisopropylethylamine (4.03 g, 31.2 mmol, 5.4 mL, 3 *eq*) at 25 °C in one portion under nitrogen. The mixture was stirred at 80 °C for 2 hours. The mixture was cooled to 25 °C and poured into ice-water (w/w = 1/1) (30 mL) and stirred for 15 min. The aqueous phase was extracted with ethyl acetate (50 mL × 3). The combined organic phase was washed with brine (50 mL × 3), dried with anhydrous sodium sulfate, filtered, and concentrated in vacuum. The residue was purified by prep thin-layer chromatography (TLC) (petroleum ether: ethyl acetate = 3:1) to afford methyl 2-[3-[4-(dimethoxymethyl)-1-piperidyl] isoxazol-5-yl]-3-methyl-butanoate (0.354 g, 1.04 mmol, 10% yield) as a yellow oil.

2-[3-[4-(Dimethoxymethyl)-1-piperidyl]isoxazol-5-yl]-3-methyl- butanoic acid (**15**)

To a solution of methyl 2-[3-[4-(dimethoxymethyl)-1-piperidyl]isoxazol-5-yl]-3-methyl- butanoate (350 mg, 1.03 mmol, 1 *eq*) in tetrahydrofuran (1 mL) and methanol (1 mL) was added lithium hydroxide (2 M, 1 mL, 1.95 *eq*) in one portion under nitrogen. The mixture was stirred at 25 °C for 1 h. The pH of the reaction mixture was adjusted to 5-6 with trifluoroacetic acid, and the mixture was concentrated under reduced pressure at 45 °C. The residue was purified by preparative High Performance Liquid chromatography (HPLC) (column: Phenomenex Synergi C18 150*25*10um; mobile phase: [water(0.225%FA)-ACN];B%: 40%-70%) to afford 2-[3-[4-(dimethoxymethyl)-1-piperidyl]isoxazol-5-yl]-3-methyl-butanoic acid (190 mg, 0.58 mmol, 56% yield) as a yellow oil.

tert-Butyl N-[(1S)-1-(4-bromophenyl)ethyl]carbamate (**16**)

To a solution of (1S)-1-(4-bromophenyl)ethanamine (24.9 g, 124.5 mmol, 1 *eq*) in tetrahydrofuran (350 mL) was added triethylamine (37.8 g, 373.4 mmol, 3 *eq*) followed by di-*tert*-butyl dicarbonate (28.5 g, 130.7 mmol, 30 mL, 1.05 *eq*) dropwise at 0 °C under nitrogen. The mixture was then stirred at 25 °C for 12 hours. The reaction mixture was concentrated under reduced pressure to remove tetrahydrofuran. Water (400 mL) was added, and the mixture was stirred for 1 minute. The aqueous phase was extracted with ethyl acetate (200 mL x 3). The combined organic phase was washed with brine (200 mL x 2), dried over anhydrous sodium sulfate, filtered, and concentrated in vacuum. The crude product was triturated with petroleum ether (250 mL). Compound *tert*-butyl N-[(1S)-1-(4-bromophenyl)ethyl]carbamate (34.5 g, 114.93 mmol, 92 % yield) was obtained as a white solid.

tert-Butyl N-[(1S)-1-[4- (4-methylthiazol-5-yl)phenyl]ethyl]carbamate (**17**)

To a solution of tert-butyl N-[(1S)-1-(4-bromophenyl)ethyl]carbamate (14.5 g, 48.30 mmol, 1 *eq*) and 4-methylthiazole (7.18 g, 72.45 mmol, 1.5 *eq*) in dimethylacetamide (15 mL) was added palladium(II) acetate (542 mg, 2.42 mmol, 0.05 *eq*) and potassium acetate (9.48 g, 96.61 mmol, 2 *eq*). The mixture was stirred at 90 °C for 12 h. Water (300 mL) was added, and the mixture was stirred for 1 minute. The aqueous phase was extracted with ethyl acetate (100 mL x 3). The combined organic phase was washed with brine (100 mL x 2), dried with anhydrous sodium sulfate, filtered, and concentrated in vacuum. The residue was purified by reverse phase C18 column chromatography [ACN/ H2O (0.5% FA) from 5% to 50%]. *tert*-Butyl N-[(1S)-1-[4- (4-methylthiazol-5-yl)phenyl]ethyl]carbamate (9.8 g, 29.85 mmol, 61% yield) was obtained as a gray solid.

(1S)-1-[4-(4-Methylthiazol-5-yl)phenyl]ethanamine (**18**)

To a solution of tert-butyl N-[(1S)-1-[4-(4-methylthiazol-5-yl)phenyl]ethyl]carbamate (1.5 g, 4.71 mmol, 1 *eq*) in dichloromethane (20 mL) was added hydrochloride acid/dioxane (4 M, 20 mL, 17 *eq*). The mixture was stirred at 25 °C for 12 hours. The reaction mixture was concentrated under reduced pressure to remove dichloromethane. The crude product was triturated with petroleum ether (100 mL). Crude (1S)-1-[4-(4-methylthiazol-5-yl)phenyl]ethanamine hydrochloride (1.1 g) was obtained as a yellow solid.

tert-Butyl (2S,4R)-4-hydroxy-2-[[(1S)-1-[4-(4-methylthiazol-5-yl)phenyl]ethyl]carbamoyl] pyrrolidine-1-carboxylate (**19**)

To a solution of (2S,4R)-1-*tert*-butoxycarbonyl-4-hydroxy-pyrrolidine-2-carboxylic acid (998 mg, 4.32 mmol, 1.1 *eq*) and O-(7-azabenzotriazol-1-yl)-N,N,N',N'-tetramethyluronium hexafluorophosphate (1.79 g, 4.71 mmol, 1.2 *eq*) in dimethylformamide (10 mL) were added (1S)-1-[4-(4-methylthiazol-5-yl)phenyl]ethanamine hydrochloride (1 g, 3.92 mmol, 1 *eq*) and diisopropylethyl amine (1.52 g, 11.77 mmol, 2.05 mL, 3 *eq*). The reaction mixture was stirred at 15°C for 0.5 hour. The reaction mixture was poured into water (20 mL) and extracted with ethyl acetate (30 mL x 3). The combined organic layers were washed with brine (50 mL x 3), dried over anhydrous sodium sulfate, filtered, and concentrated under reduced pressure. The residue was purified by silica gel column chromatography (petroleum ether: ethyl acetate from 100:1 to 30:1). *tert*-Butyl (2S,4R)-4-hydroxy-2-[[(1S)-1-[4-(4-methylthiazol-5-yl)phenyl]ethyl]carbamoyl] pyrrolidine-1-carboxylate (1.2 g, 2.78 mmol, 70% yield) was obtained as a white solid.

(2S,4R)-4-hydroxy-N-[(1S)-1-[4-(4-methylthiazol-5-yl)phenyl]ethyl]pyrrolidine-2-carboxamide (**20**)

To a solution of *tert*-butyl (2S,4R)-4-hydroxy-2-[[(1S)-1-[4-(4-methylthiazol-5-yl) phenyl]ethyl]carbamoyl]pyrrolidine-1-carboxylate (1 g, 2.32 mmol, 1 *eq*) in dichloromethane (10 mL) was added hydrochloric acid (2.5 M in dioxane, 5 mL, 5.4 *eq*). The reaction mixture was stirred at 15 °C for 0.5 hour. The reaction mixture was concentrated under reduced pressure. (2S,4R)-4-hydroxy-N-[(1S)-1-[4-(4-methylthiazol-5-yl)phenyl]ethyl]pyrrolidine-2-carboxamide hydrochloride (800 mg, 2.17 mmol, 93% yield) was obtained as a colorless oil.

(2S,4R)-1-[2-[3-[4-(dimethoxymethyl)-1-piperidyl]isoxazol-5-yl]-3-methyl-butanoyl]-4-hydroxy-N-[(1S)-1-[4-(4-methylthiazol-5-yl)phenyl]ethyl]pyrrolidine-2-carboxamide (**21**)

To a mixture of 2-[3-[4-(dimethoxymethyl)-1-piperidyl]isoxazol-5-yl]-3-methyl-butanoic acid (190 mg, 0.58 mmol, 1 *eq*), *N,N*-diisopropylethylamine (225 mg, 1.75 mmol, 0.3 mL, 3 *eq*) and (2S,4R)-4-hydroxy-N-[(1S)-1-[4-(4-methylthiazol-5-yl)phenyl]ethyl]pyrrolidine-2-carboxamide hydrochloride (257 mg, 0.70 mmol, 1.2 *eq*) in *N,N*-dimethylformamide (10 mL) was added O-(7-azabenzotriazol-1-yl)-N,N,N',N'-tetramethyluronium hexafluorophosphate (288 mg, 0.76 mmol, 1.3 *eq*) in one portion at 0 °C under nitrogen. The mixture was stirred at 25 °C for 1 h. The mixture was poured into ice-water (w/w = 1/1) (30 mL) and stirred for 15 min. The aqueous phase was extracted with ethyl acetate (30 mL x 3). The combined organic phase was washed with brine (30 mL x 3), dried with sodium sulfate, filtered, and concentrated in vacuum. The residue was purified by silica gel chromatography (dichloromethane: methanol = 10:1) to afford (2S,4R)-1-[2-[3-[4-(dimethoxymethyl)-1-piperidyl]isoxazol-5-yl]-3-methyl-butanoyl]-4-hydroxy-N-[(1S)-1-[4-(4-methylthiazol-5-yl)phenyl]ethyl]pyrrolidine-2-carboxamide (208 mg, 0.32 mmol, 55% yield) as a yellow oil.

(2S,4R)-1-[(2S)-2-[3-[4-(dimethoxymethyl)-1-piperidyl]isoxazol-5-yl]-3-methyl-butanoyl]-4-hydroxy-N-[(1S)-1-[4-(4-methylthiazol-5-yl)phenyl]ethyl]pyrrolidine-2-carboxamide (**22**) and (2S,4R)-1-[(2R)-2-[3-[4-(dimethoxymethyl)-1-piperidyl]isoxazol-5-yl]-3-methyl-butanoyl]-4-hydroxy-N-[(1S)-1-[4-(4-methylthiazol-5-yl)phenyl]ethyl]pyrrolidine-2-carboxamide (**23**)
(2S,4R)-1-[2-[3-[4-(dimethoxymethyl)-1-piperidyl]isoxazol-5-yl]-3-methyl-butanoyl]-4-hydroxy-N-[(1S)-1-[4-(4-methylthiazol-5-yl)phenyl]ethyl]pyrrolidine-2-carboxamide (200 mg, 0.31 mmol, 1 *eq*) was purified by chiral SFC (column: DAICEL CHIRALPAK AD (250mm*30mm,10um); mobile phase: [0.1%NH4OH/ IPA]; B%: 45%) to produce (2S,4R)-1-[(2S)-2-[3-[4-(dimethoxymethyl)-1-piperidyl]isoxazol-5-yl]-3-methyl-butanoyl]-4-hydroxy-N-[(1S)-1-[4-(4-methylthiazol-5-yl)phenyl]ethyl]pyrrolidine-2-carboxamide (84 mg, 0.12 mmol, 42% yield) (analytical retention time 1.88 min) as a yellow oil and (2S,4R)-1-[(2R)-2-[3-[4-(dimethoxymethyl)-1-piperidyl]isoxazol-5-yl]-3-methyl-butanoyl]-4-hydroxy-N-[(1S)-1-[4-(4-methylthiazol-5-yl)phenyl]ethyl]pyrrolidine-2-carboxamide (90 mg, 0.13 mmol, 45% yield) (analytical retention time 2.23 min) as a yellow oil.

(2S,4R)-1-[(2R)-2-[3-(4-formyl-1-piperidyl)isoxazol-5-yl]-3-methyl-butanoyl]-4-hydroxy-N-[(1S)-1-[4-(4-methylthiazol-5-yl)phenyl]ethyl]pyrrolidine-2-carboxamide (**24**)
To a solution of (2S,4R)-1-[(2R)-2-[3-[4-(dimethoxymethyl)-1-piperidyl]isoxazol-5-yl]-3- methyl-butanoyl]-4-hydroxy-N-[(1S)-1-[4-(4-methylthiazol-5-yl)phenyl]ethyl]pyrrolidine-2-carboxamide (80 mg, 0.13 mmol, 1 *eq*) in tetrahydrofuran (2 mL) was added sulfuric acid solution (1 M, 2 mL, 16 *eq*). The reaction mixture was stirred at 50 °C for 5 hours. To the mixture was added saturated sodium bicarbonate solution (10 mL), and the mixture was extracted with ethyl acetate (20 mL x 3). The combined organic phase was washed with brine (30 mL), dried over sodium sulfate, filtered, and concentrated in vacuum. The crude (2S,4R)-1-[(2R)-2-[3-(4-formyl-1-piperidyl)isoxazol-5-yl]-3-methyl-butanoyl]-4-hydroxy-N-[(1S)-1-[4-(4-methylthiazol-5-yl)phenyl]ethyl]pyrrolidine-2-carboxamide (68 mg, 0.11 mmol, 91% yield) was obtained as a light yellow solid and used directly in the next step.

(2S,4R)-1-((2R)-2-(3-(4-((4-((1r,3r)-3-((4-(3-(3-Amino-6-(2-hydroxyphenyl)pyridazin-4-yl)-3,8-diazabicyclo[3.2.1]octan-8-yl)pyridin-2-yl)oxy)cyclobutoxy)piperidin-1-yl)methyl)piperidin-1-yl)isoxazol-5-yl)-3-methylbutanoyl)-4-hydroxy-N-((S)-1-(4-(4-methylthiazol-5-yl)phenyl)ethyl)pyrrolidine-2-carboxamide (**A947**)
To a solution of 2-(6-amino-5-(8-(2-((1r,3r)-3-(piperidin-4-yloxy)cyclobutoxy)pyridin-4-yl)-3,8-diazabicyclo[3.2.1]octan-3-yl)pyridazin-3-yl)phenol hydrochloride (365 mg, 0.59 mmol, 1 *eq*) in methanol (20 mL) was added sodium acetate (145 mg, 1.77 mmol, 3 *eq*). To the mixture were then added (2S,4R)-1-[(2R)-2-[3-(4-formyl-1-piperidyl)isoxazol- 5-yl]-3-methyl-butanoyl]-4-hydroxy-N-[(1S)-1-[4-(4-methylthiazol-5-yl)phenyl]ethyl]pyrrolidine-2-carboxamide (350 mg, 0.59 mmol, 1 *eq*) and acetic acid (4 mg, 0.06 mmol, 0.1 *eq*). The mixture was stirred at 25 °C for 0.5 hour, after which sodium cyanoborohydride (185 mg, 2.95 mmol, 5 *eq*) was added. The mixture was stirred at 25 °C for 2 hours. The reaction mixture was concentrated under reduced pressure. The residue was purified by prep-HPLC (column: Phenomenex Synergi Max-RP 150*50 mm*10 um; mobile phase: [water (0.2% FA) - ACN]; B%: 80% - 38%). (2S,4R)-1-((2R)-2-(3-(4-((4-((1r,3r)-3-((4-(3-(3-Amino-6-(2-hydroxyphenyl)pyridazin-4-yl)-3,8-diazabicyclo[3.2.1]octan-8-yl)pyridin-2-yl)oxy)cyclobutoxy)piperidin-1-yl)methyl)piperidin-1-yl)isoxazol-5-yl)-3-methylbutanoyl)-4-hydroxy-N-((S)-1-(4-(4-methylthiazol-5-yl)phenyl)ethyl)pyrrolidine-2-carboxamide formate (266 mg, 0.23 mmol, 39% yield) was obtained as a white solid. **^{1}H NMR** (400MHz, DMSO-d_6) δ: 9.02 - 8.78 (m, 1H), 8.30 (br d, *J*=7.2 Hz, 1H), 8.05 (s, 1H), 7.81 (br d, *J*=7.6 Hz, 1H), 7.67 (br d, *J*=5.6 Hz, 1H), 7.45 - 7.25 (m, 5H), 7.12 (br t, *J*=7.6 Hz, 1H), 6.83 - 6.67 (m, 2H), 6.55 - 6.50 (m, 1H), 6.18 - 6.10 (m, 2H), 6.09 - 5.93 (m, 2H), 5.25 - 5.15 (m, 1H), 5.13 - 4.97 (m, 1H), 4.96 - 4.86 (m, 1H), 4.50 (br

s, 2H), 4.40 - 4.25 (m, 3H), 3.77 - 3.45 (m, 4H), 3.27 - 3.12 (m, 6H), 3.06 - 2.72(m, 8H), 2.46 (s, 3H), 2.38- 2.05 (m, 8H), 2.03 - 1.62 (m, 11H), 1.48 - 1.34 (m, 3H), 1.22 (br d, *J*=12.0 Hz, 2H), 1.02 - 0.90 (m, 3H), 0.88 - 0.73 (m, 3H). (ESI) m/z calculated for $C_{61}H_{76}N_{12}O_7S + H^+$ $[M+H]^+$: 1121.6. Found: 1121.7.

tert-Butyl (2S,4S)-4-hydroxy-2-[[(1S)-1-[4-(4-methylthiazol-5-yl)phenyl] ethyl]carbamoyl]pyrrolidine-1-carboxylate (**25**)

A mixture of (1S)-1-[4-(4-methylthiazol-5-yl)phenyl]ethanamine (2.4 g, 9.42 mmol, 1 *eq*, hydrochloride), (2S,4S)-1-tert-butoxycarbonyl-4-hydroxy-pyrrolidine-2-carboxylic acid (2.18 g, 9.42 mmol, 1 *eq*), O-(7-azabenzotriazol-1-yl)-*N,N,N',N'*-tetramethyluronium hexafluorophosphate (4.30 g, 11.30 mmol, 1.2 *eq*), and triethylamine (2.86 g, 28.26 mmol, 3.93 mL, 3 *eq*) in *N,N*-dimethylformamide (40 mL) was degassed and purged with nitrogen 3 times. The mixture was then stirred at 25 °C for 1 h under nitrogen. The reaction mixture was diluted with water (200 mL) and extracted with ethyl acetate (200 mL × 2). The combined organic phase was washed with brine (50 mL), dried over anhydrous sodium sulfate, filtered, and concentrated in vacuum. The residue was purified by preparative HPLC (column: Phenomenex luna C18 250*50 mm*10 um; mobile phase: [water (0.225% FA) - ACN]; B%: 25% - 55%). *tert*-Butyl (2S,4S)-4-hydroxy-2-[[(1S)-1-[4-(4-methylthiazol-5-yl)phenyl] ethyl]carbamoyl]pyrrolidine-1-carboxylate (3.5 g, 8.11 mmol, 86% yield) was obtained as a white solid.

(2S,4S)-1-((2R)-2-(3-(4-((4-((1r,3r)-3-((4-(3-(3-amino-6-(2-hydroxyphenyl)pyridazin-4-yl)-3,8-diazabicyclo[3.2.1]octan-8-yl)pyridin-2-yl)oxy)cyclobutoxy)piperidin-1-yl)methyl)piperidin-1-yl)isoxazol-5-yl)-3-methylbutanoyl)-4-hydroxy-N-((S)-1-(4-(4-methylthiazol-5-yl)phenyl)ethyl)pyrrolidine-2-carboxamide (**A857**)
(2S,4S)-1-((2R)-2-(3-(4-((4-((1r,3r)-3-((4-(3-(3-amino-6-(2-hydroxyphenyl)pyridazin-4-yl)-3,8-diazabicyclo[3.2.1]octan-8-yl)pyridin-2-yl)oxy)cyclobutoxy)piperidin-1-yl)methyl)piperidin-1-yl)isoxazol-5-yl)-3-methylbutanoyl)-4-hydroxy-N-((S)-1-(4-(4-methylthiazol-5-yl)phenyl)ethyl)pyrrolidine-2-carboxamide was prepared from *tert*-butyl (2S,4S)-4-hydroxy-2-[[(1S)-1-[4-(4-methylthiazol-5-yl)phenyl] ethyl]carbamoyl]pyrrolidine-1-carboxylate (**25**) according to the Schemes 6 and 7 using procedures described for the preparation of **A947** from intermediate **20**. **^{1}H NMR** (400 MHz, DMSO-*d*$_6$) *δ:* 9.02 - 8.99 (m, 1H), 8.39 (d, *J*=7.6 Hz, 1H), 8.14 (s, 1H), 8.03 - 7.89 (m, 1H), 7.84 - 7.73 (m, 1H), 7.52 - 7.36 (m, 5H), 7.23 (t, *J*=7.6 Hz, 1H), 6.95 - 6.83 (m, 2H), 6.52 - 6.49 (m, 1H), 6.21 - 6.12 (m, 2H), 5.99 (s, 2H), 5.37 - 5.18 (m, 2H), 4.94 (quin, *J*=7.2 Hz, 1H), 4.50 (br s, 2H), 4.38 - 4.24 (m, 2H), 4.20 (br d, *J*=5.6 Hz, 1H), 3.75 - 3.59 (m, 4H), 3.59 - 3.50 (m, 2H), 3.29 - 3.12 (m, 4H), 3.08 - 2.72 (m, 8H), 2.46 (s, 3H), 2.40 - 2.10 (m, 9H), 2.02 - 1.61 (m, 10H), 1.48 - 1.34 (m, 3H), 1.31 - 1.10 (m, 2H), 1.01 - 0.91 (m, 3H), 0.89 - 0.74 (m, 3H). (ESI) m/z calculated for $C_{61}H_{76}N_{12}O_7S + H^+$ $[M+H]^+$: 1121.6. Found: 1121.8.

(2S,4R)-1-((2R)-2-(3-(4-((4-((1r,3r)-3-((4-(3-(3-amino-6-phenylpyridazin-4-yl)-3,8-diazabicyclo[3.2.1]octan-8-yl)pyridin-2-yl)oxy)cyclobutoxy)piperidin-1-yl)methyl)piperidin-1-yl)isoxazol-5-yl)-3-methylbutanoyl)-4-hydroxy-N-((S)-1-(4-(4-methylthiazol-5-yl)phenyl)ethyl)pyrrolidine-2-carboxamide (**A858**)
(2S,4R)-1-((2R)-2-(3-(4-((4-((1r,3r)-3-((4-(3-(3-amino-6-phenylpyridazin-4-yl)-3,8-diazabicyclo[3.2.1]octan-8-yl)pyridin-2-yl)oxy)cyclobutoxy)piperidin-1-yl)methyl)piperidin-1-yl)isoxazol-5-yl)-3-methylbutanoyl)-4-hydroxy-N-((S)-1-(4-(4-methylthiazol-5-yl)phenyl)ethyl)pyrrolidine-2-carboxamide (**A858**) was prepared from *tert*-butyl 4-((1r,3r)-3-((4-(3-(3-amino-6-chloropyridazin-4-yl)-3,8-diazabicyclo[3.2.1]octan-8-yl)pyridin-2-yl)oxy)cyclobutoxy)piperidine-1-carboxylate (**9**) according to the Scheme 8 using procedures described for the preparation of **A947**. **^{1}H NMR** (400 MHz, DMSO-*d*$_6$) δ: 8.97 (s, 1H), 8.37 (d, *J* = 7.6 Hz, 1H), 8.12 (s, 1H), 7.95 (d, *J* = 7.2 Hz, 1H), 7.74 (d, *J* = 6.0 Hz, 1H), 7.46 - 7.39 (m, 3H), 7.39 - 7.31 (m, 3H), 7.24 (s, 1H), 6.61 - 6.41 (m, 2H), 6.21 - 5.96 (m, 2H), 5.71 (s, 2H), 5.27 - 5.13 (m, 1H), 5.09 (br d, *J* = 2.0 Hz, 1H), 4.89 (br t, *J* = 7.2 Hz, 1H), 4.46 (br s, 2H), 4.34 (t, *J* = 8.0 Hz, 1H), 4.30 - 4.22 (m, 2H), 3.69 (br dd, *J* = 4.4, 10.4 Hz, 1H), 3.61 (br d, *J* = 12.0 Hz, 2H), 3.57 - 3.52 (m, 1H), 3.41 (br d, *J* = 2.8 Hz, 4H), 3.19 (br d, *J* = 11.2 Hz, 3H), 2.92 (br d, *J* = 11.2 Hz, 4H), 2.78 - 2.69 (m, 2H), 2.62 - 2.54 (m, 1H), 2.44 (s, 4H), 2.33 - 2.25 (m, 4H), 2.22 - 2.10 (m, 3H), 2.00 (br s, 1H), 1.96 - 1.90 (m, 2H), 1.89 - 1.82 (m, 2H), 1.81 - 1.66 (m, 4H), 1.64 - 1.50 (m, 2H), 1.47 - 1.30 (m, 3H), 1.26 - 1.07 (m, 2H), 1.01 - 0.86 (m, 3H), 0.77 (d, *J* = 6.8Hz, 3H). (ESI) m/z calculated for $C_{61}H_{76}N_{12}O_6S + H^+$ $[M+H]^+$: 1105.6. Found: 1105.6.

(2S,4R)-4-hydroxy-1-[(2R)-2-(3-methoxy-1,2-oxazol-5-yl)-3-methylbutanoyl]-N-[[4-(4-methyl-1,3-thiazol-5-yl)phenyl]methyl]pyrrolidine-2-carboxamide (**A2702**)
Into a 50-mL round-bottom flask was placed a solution of (2S,4R)-4-hydroxy-N-[[4-(4-methyl-1,3-thiazol-5-yl)phenyl]methyl]pyrrolidine-2-carboxamide hydrochloride **31** [prepared as described for the intermediate **20** above]

(200.0 mg, 0.57 mmol, 1 eq) in N,N-dimethylformamide (10 mL). N,N,N',N'-Tetramethyl-O-(7-azabenzotriazol-1-yl)uronium hexafluorophospate (450 mg, 1.18 mmol, 1.2 eq), N,N-diisopropylethylamine (520 mg, 4.02 mmol, 4 eq), 2-(3-methoxy-1,2-oxazol-5-yl)-3-methylbutanoic acid[2] (317 mg, 1.59 mmol, 1 eq) were added. The resulting solution was stirred for 2 hours at room temperature. The reaction was then quenched by the addition of 20 mL of water and extracted with ethyl acetate (100 mL x 2). The organic layers were combined and concentrated under reduced pressure. The residue was initially purified on a silica gel column eluting with ethyl acetate. The product was purified by chiral prep HPLC (column, CHIRALPAK ID-03, 2.0cm I.D*25cm L(5um); mobile phase, hexane/ IPA (hold 50% IPA; 20 min). (2S,4R)-4-Hydroxy-1-[(2R)-2-(3-methoxy-1,2-oxazol-5-yl)-3-methylbutanoyl]-N-[[4-(4-methyl-1,3-thiazol-5-yl)phenyl]methyl]pyrrolidine-2-carboxamide (20.4 mg, 0.04 mmol, 8% yield) was produced as a white solid. **^{1}H NMR** (400 MHz, methanol-*d$_4$*): δ 8.90 (s, 1H), 7.51-7.41 (m, 4H), 5.99 (s, 1H), 4.62-4.48 (m, 4H), 3.96-3.89 (m, 4H), 3.79-3.73 (m, 2H), 2.50 (d, J = 3.8 Hz, 3H), 2.46- 2.29 (m, 1H), 2.31-2.18 (m, 1H), 2.09-2.01 (m, 1H), 1.07 (d, J = 6.7 Hz, 3H), 0.93 (d, J = 6.7 Hz, 3H). (ESI) m/z calculated for $C_{25}H_{30}N_4O_5S + H^+$ $[M+H]^+$: 499.2. Found: 499.0.

1. Crew, A. et al. Tetrahydronaphthalene and tetrahydroisoquinoline derivatives as estrogen receptor degraders and their preparation. PCT Int. Appl. (2018), WO 2018102725.
2. Yimin, Q. et al. Preparation of bifunctional PROTAC compounds and methods for the enhanced degradation of targeted bromodomain-containing proteins. PCT Int. Appl. (2017), WO 2017030814.

Scheme 1. Synthesis of Intermediate **5**

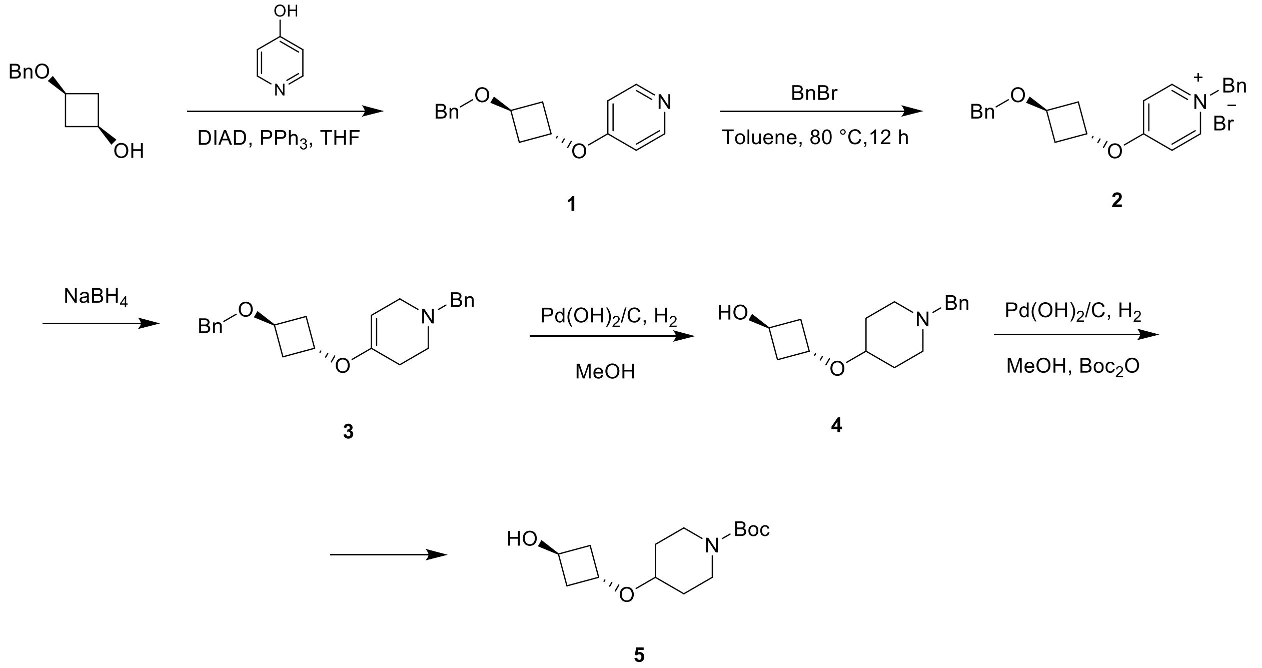

Scheme 2. Synthesis of Intermediate **11**

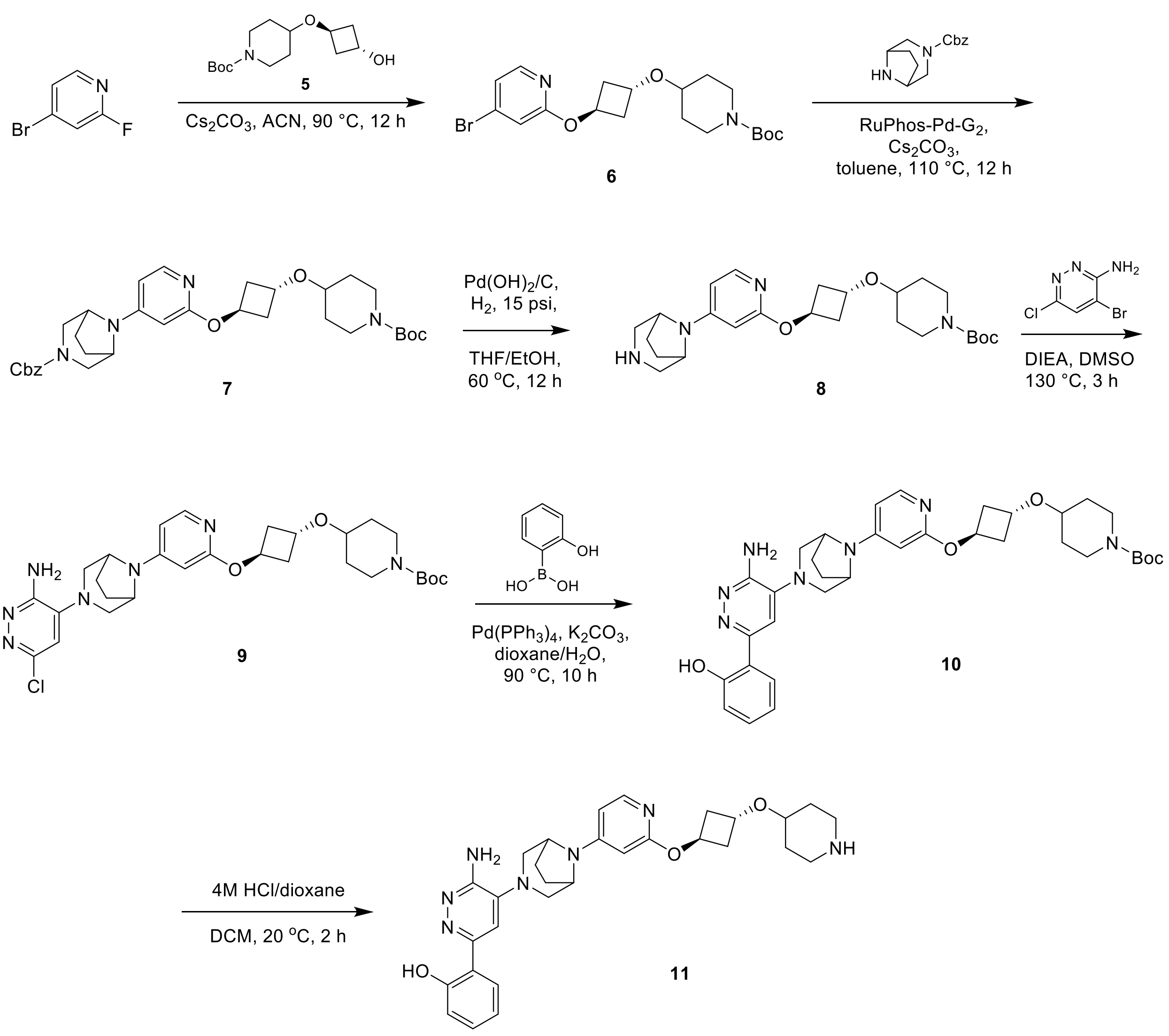

Scheme 3. Synthesis of intermediate **20**

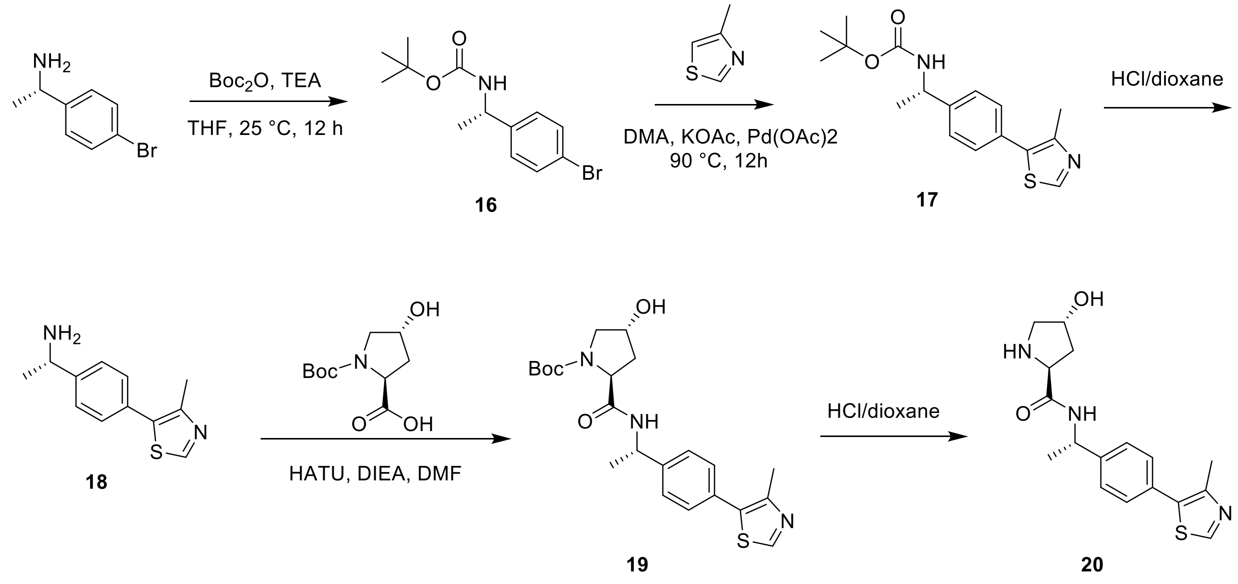

Scheme 4. Synthesis of intermediate **24**

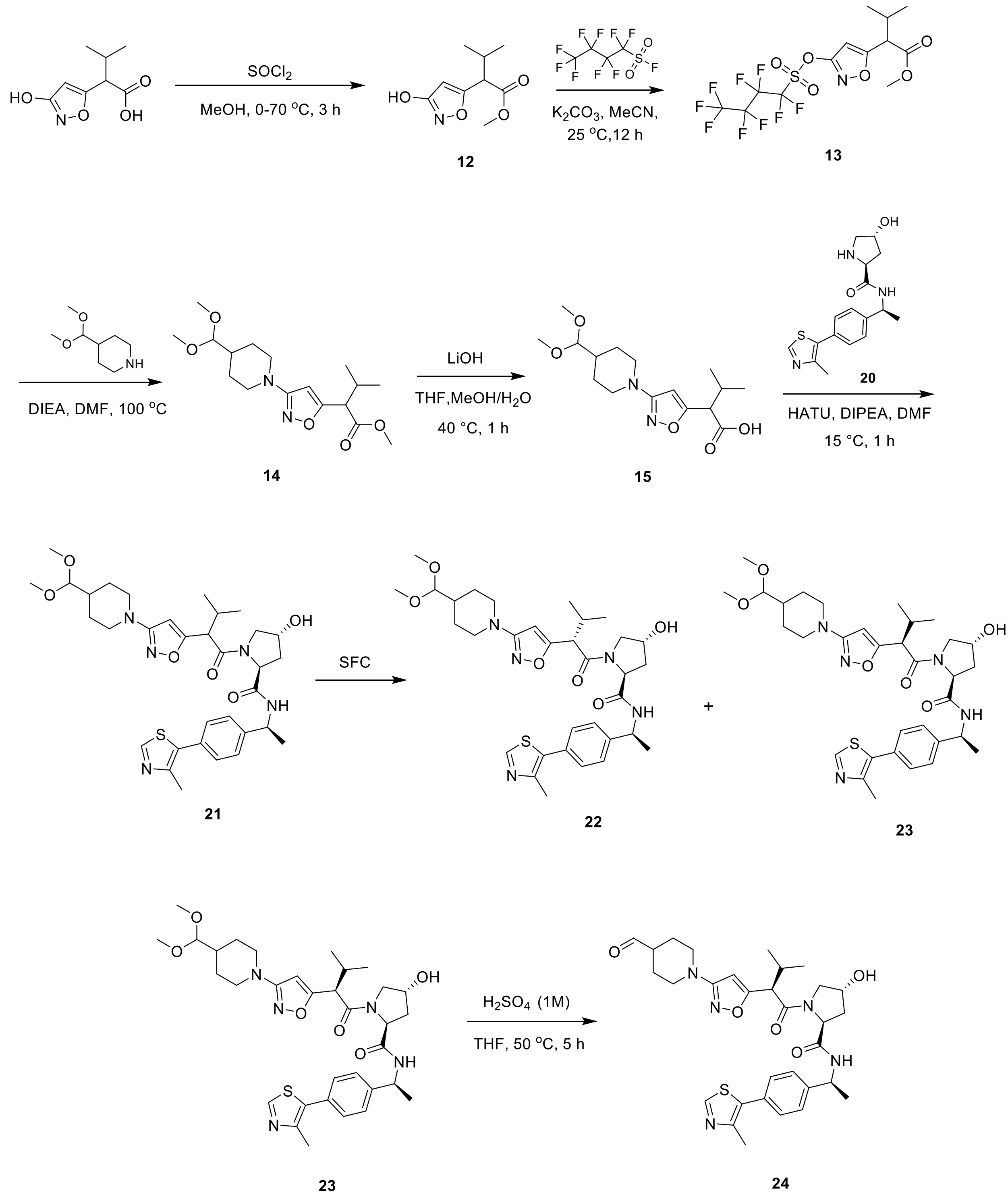

Scheme 5. Synthesis of **A947**

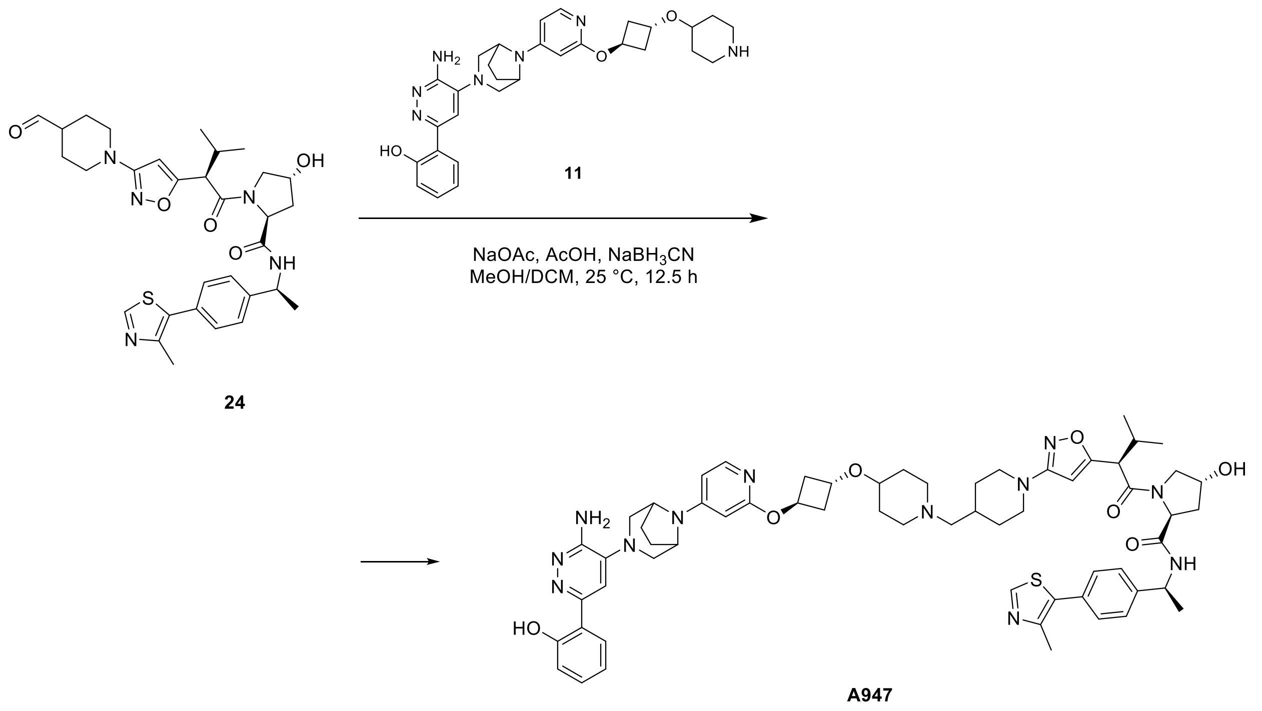

Scheme 6. Synthesis of intermediate **29**

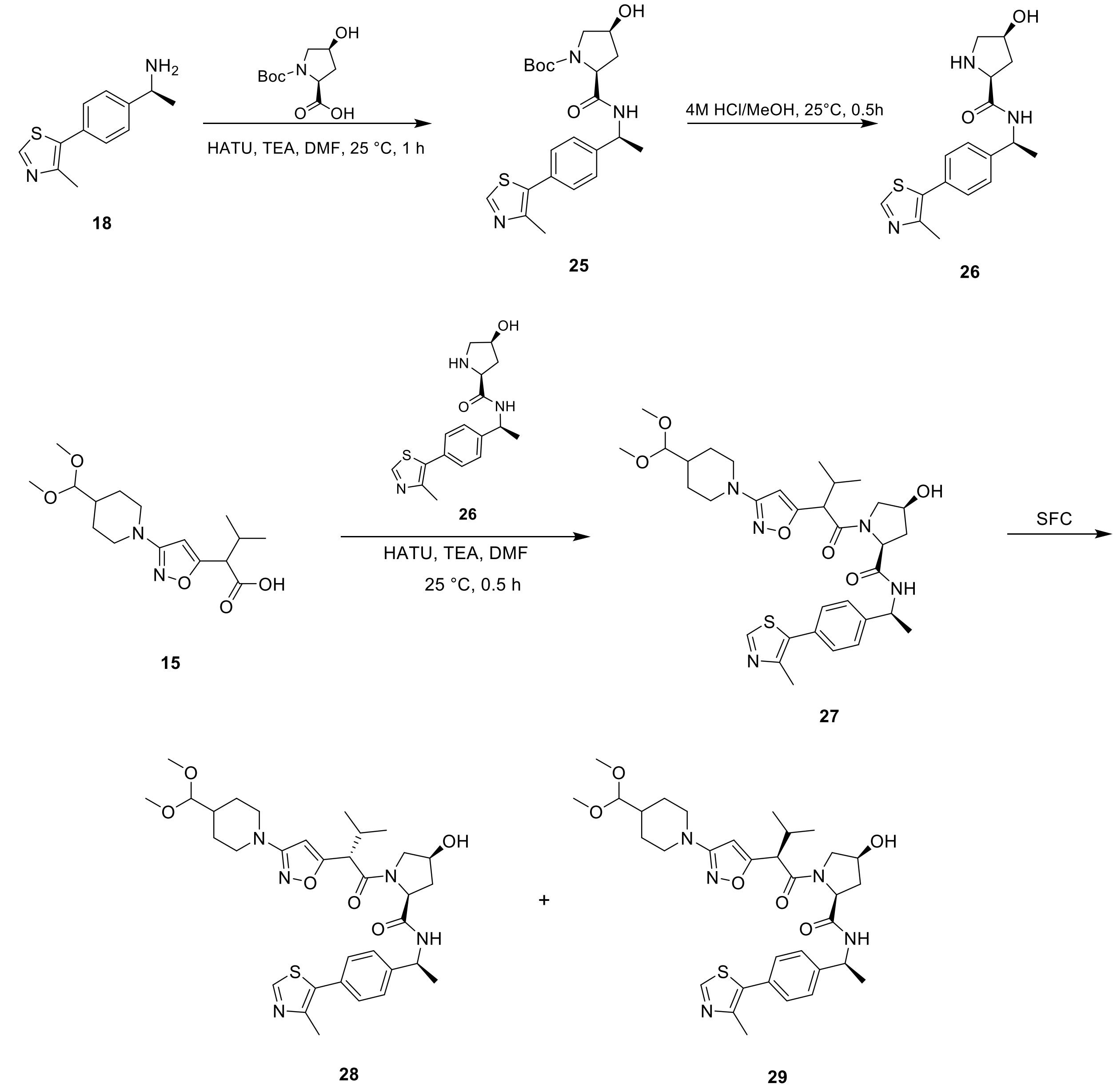

Scheme 7. Synthesis of **A857**

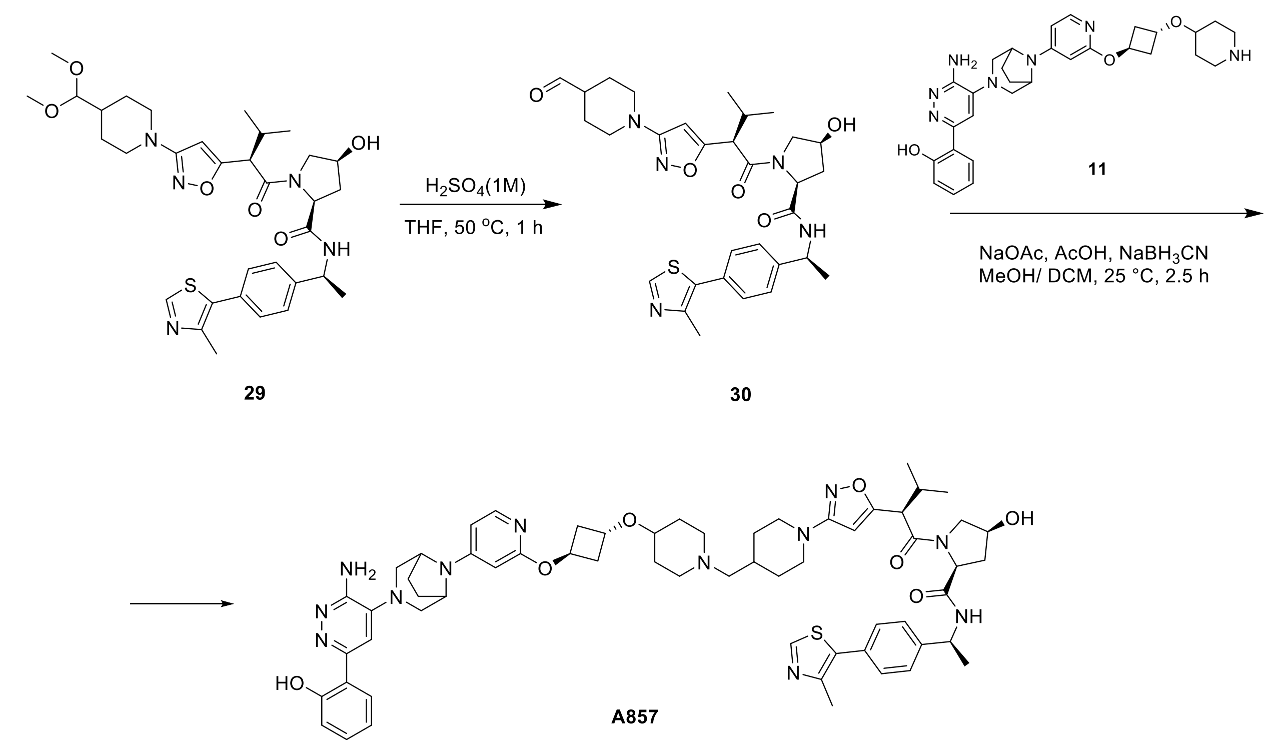

Scheme 8. Synthesis of **A858**

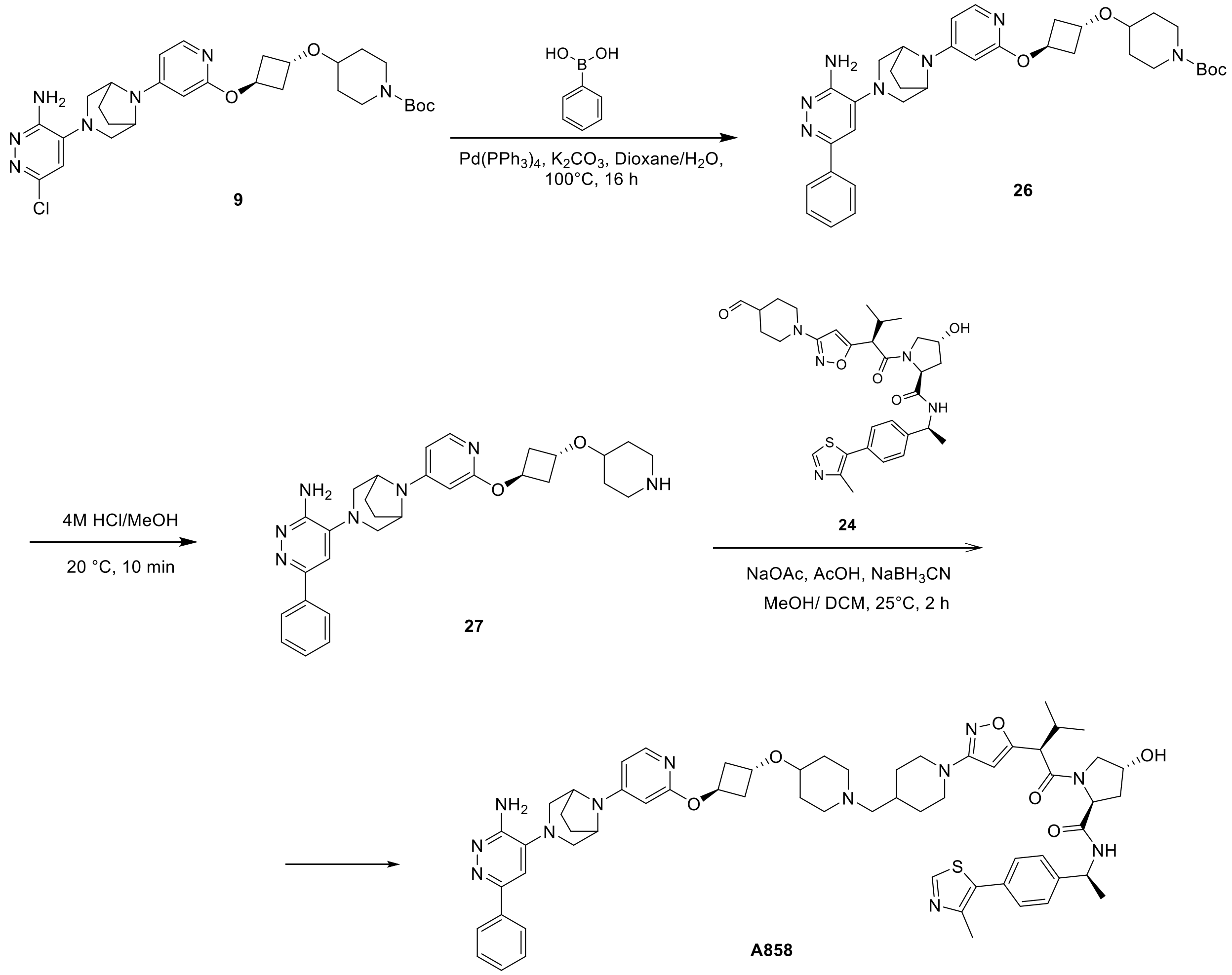

Scheme 9. Synthesis of **A2702**

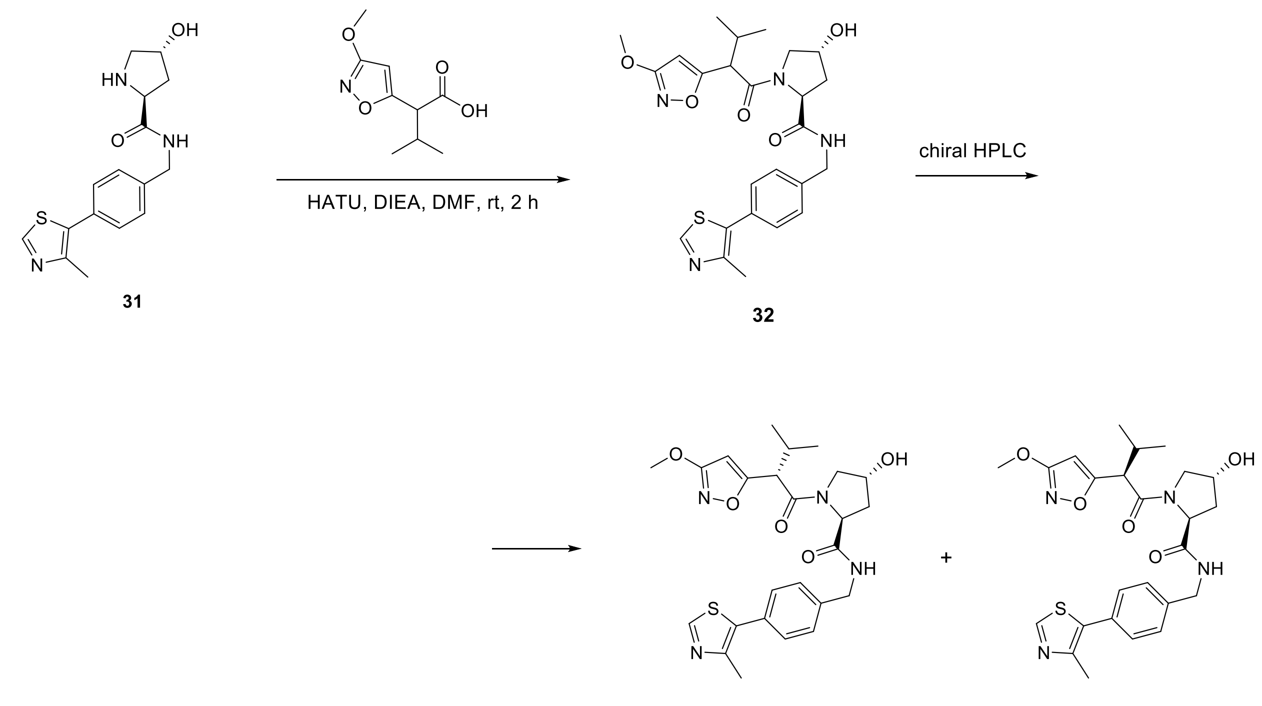

33 **A2702**
