# Supplementary material for: Selective PROTAC-mediated degradation of SMARCA2 is efficacious in SMARCA4 mutant cancers
Source: Nat Commun. 2022 Nov 10;13:6814. doi: 10.1038/s41467-022-34562-5 (PMC9649729; doi:10.1038/s41467-022-34562-5)
Supplement: Supplementary file 3 — Description of Additional Supplementary Files [file 41467_2022_34562_MOESM3_ESM.pdf]

Description of Additional Supplementary Files

Supplementary Data 1: Quantitative di-glycine reminant profiling by mass spectrometry to evaluate changes in the ubiquitylome in SW1573 cells following A947 treatment.

Supplementary Data 2: Mass spectrometry to assess the impact of A947 treatment in SW1573 cells on the global proteome.

Supplementary Data 3: Characterization of NSCLC cell lines used in the manuscript.

Supplementary Data 4: Gene expression changes determined by RNAseq in HCC2302 and HCC515 cells following treatment with A947 and/or inducible knockdown of SMARCA2.

Supplementary Data 5: Assessment of pharmacologic combinations of A947 with 723 experimental agents across 4 SMARCA4-mutant NSCLC cell line models
